# Identification of *Camellia oleifera* WRKY transcription factor genes and functional characterization of CoWRKY78

**DOI:** 10.3389/fpls.2023.1110366

**Published:** 2023-03-09

**Authors:** Jingbin Li, Chaowei Xiong, Dong Ruan, Wei Du, He Li, Chengjiang Ruan

**Affiliations:** Key Laboratory of Biotechnology and Bioresources Utilization-Ministry of Education, Institute of Plant Resources, Dalian Minzu University, Dalian, China

**Keywords:** *Camellia oleifera*, anthracnose, *CoWRKY* genes, gene expression patterns, ROS homeostasis

## Abstract

*Camellia oleifera* Abel is a highly valued woody edible oil tree, which is endemic to China. It has great economic value because *C. oleifera* seed oil contains a high proportion of polyunsaturated fatty acids. *C. oleifera* anthracnose caused by *Colletotrichum fructicola*, poses a serious threat to *C. oleifera* growth and yield and causes the benefit of the *C. oleifera* industry to suffer directly. The WRKY transcription factor family members have been widely characterized as vital regulators in plant response to pathogen infection. Until now, the number, type and biological function of *C. oleifera WRKY* genes are remains unknown. Here, we identified 90 C*. oleifera* WRKY members, which were distributed across 15 chromosomes. *C. oleifera WRKY* gene expansion was mainly attributed to segmental duplication. We performed transcriptomic analyses to verify the expression patterns of *CoWRKYs* between anthracnose-resistant and -susceptible cultivars of *C. oleifera*. These results demonstrated that multiple candidate *CoWRKY*s can be induced by anthracnose and provide useful clues for their functional studies. *CoWRKY78*, an anthracnose-induced *WRKY* gene, was isolated from *C. oleifera*. It was significantly down-regulated in anthracnose-resistant cultivars. Overexpression of *CoWRKY78* in tobacco markedly reduced resistance to anthracnose than WT plants, as evidenced by more cell death, higher malonaldehyde content and reactive oxygen species (ROS), but lower activities of superoxide dismutase (SOD), peroxidase (POD), as well as phenylalanine ammonia-lyase (PAL). Furthermore, the expression of multiple stress-related genes, which are associated with ROS-homeostasis (*NtSOD* and *NtPOD*), pathogen challenge (*NtPAL*), and pathogen defense (*NtPR1*, *NtNPR1*, and *NtPDF1.2*) were altered in the *CoWRKY78*-overexpressing plants. These findings increase our understanding of the *CoWRKY* genes and lay the foundation for the exploration of anthracnose resistance mechanisms and expedite the breeding of anthracnose-resistant *C. oleifera* cultivars.

## 1 Introduction

*Camellia oleifera* Abel is an evergreen small tree or shrub in the family Theaceae. Together with coconut (*Cocos nucifera*), olive (*Canarium album*), and palm (*Trachycarpus fortunei*), they are called the world’s four major woody oil tree species (Zhang et al., 2021). As one kind of oil-rich seed tree, *C. oleifera* has great economic value, nutritional and medicinal value because its seed oil is rich in unsaturated fatty acids and natural bioactive ingredients (Ye et al., 2021). In the past several years, most *C. oleifera* studies have focused mainly on oil extraction technology (Zhang et al., 2019a), self-incompatibility (Zhou et al., 2020), seed oil biosynthesis (Zhang et al., 2021), seed development (Wu et al., 2022), and fruit development (He et al., 2022a). Specifically, with ecological value, *C. oleifera* can not only live in the cold climate (Wu et al., 2020), but also grow well in drought and barren soil (He et al., 2022b). However, *C. oleifera* is vulnerable to a number of fungal and bacterial infections, which seriously threaten the healthy and sustainable development of *C. oleifera* industry. *C. oleifera* anthracnose, which is caused by *Colletotrichum fructicola*, is the primary disease of *C. oleifera*, and seriously affects yield and tea-oil quality (Zhang et al., 2019b). Chemical pesticides can prevent *C. oleifera* anthracnose, but this may induce problems such as chemical residues on the tree, fungicide resistance, and environmental pollution. Therefore, selecting resistance genes to develop resistant cultivars would be an effective method to manage diseases. Nevertheless, limited knowledge exists regarding the molecular mechanisms underlying anthracnose resistance.

Plants have evolved sophisticated defense mechanism to defend themselves from various pathogenic diseases (Kang et al., 2018). This process requires different types of transcription factors (TFs), which play essential roles in transcriptional regulation (Amorim et al., 2017; Yan et al., 2022). WRKY proteins constitute one of the largest TF families in land plants, and each member has one or two conserved WRKY domains at the N-terminus region, followed closely by a Cys_2_HisCys-type or a Cys_2_His_2_-type zinc-finger domain at the C-terminus region (Lim et al., 2022). WRKY TFs can specifically recognize the W-box, with sequence TTGAC/T within the target genes’ promoter regions (Ciolkowski et al., 2008). WRKY TF families can comprise 3 groups (Du et al., 2022). The members in the group I have two WRKY structural domains with Cys_2_His_2_-type motifs. Furthermore, members in group II or group III that have one WRKY structural domain with Cys_2_His_2_-type or Cys_2_HisCys-type motif. Lots of evidence showing that WRKY TFs plays a central role involved in disease resistance in the plant through a variety of pathways. In *Arabidopsis thaliana*, overexpression of *AtWRKY75* enhanced plant resistance to *Sclerotinia sclerotiorum* and increased expression of *PDF1.2* (Chen et al., 2013). Meanwhile, transgenic *A. thaliana* overexpressing *AtWRKY70* showed resistance against *Pseudomonas syringae* and increased the *PR* gene expression level (Li et al., 2004). In *Oryza sativa*, overexpression of *OsWRKY30* (Peng et al., 2012) or *OsWRKY45* (Huangfu et al., 2016) led to increased resistance against *Magnaporthe grisea*. Moreover, *ShWRKY41* (Lian et al., 2022), *CmWRKY15-1* (Bi et al., 2021), *PlWRKY65* (Wang et al., 2020), and *FaWRKY25* (Jia et al., 2020) have been shown to function as negative or positive regulators involved in the plant defense response to various pathogen infection. In woody plants, overexpression of *WRKY60* in *Populus tomentosa* led to enhanced resistance to *Dothiorella gregaria* and increased *PR* gene expression (Ye et al., 2014). As well, transgenic poplars overexpressing *PtrWRKY18* or *PtrWRKY35* showed enhanced resistance against *Melampsora* (Jiang et al., 2017). In addition, *RcWRKY41*, was inferred as a candidate regulator in response to *Botrytis cinerea* infection in roses (Liu et al., 2019). So far, 72 and 109 WRKY TFs have already been identified in *Arabidopsis* or rice (Eulgem and Somssich, 2007; Ross et al., 2007). Furthermore, 104 and 80 WRKY members were identified in the poplar and grape genome (He et al., 2012; Zhang and Feng, 2014). However, systematic information on WRKYs in *C. oleifera* was unclear.

WRKY TFs are of great importance in plant-pathogen interactions (Li et al., 2015). However, there have not been any reports investigating the expression pattern and function of *WRKY* genes directly involved in *C. oleifera*-*C. fructicola* interaction. The genome sequence of *C. oleifera* has recently been completed (Lin et al., 2022). Key metabolites involved in *C. oleifera* against anthracnose have been investigated through integrated transcriptome and metabolome analysis (Yang et al., 2022a). Overexpression of *CoDFR* in *Nicotiana tabacum* L. increased salicylic acid content as well as promoted the accumulation of flavonoids and thereby increased resistance to anthracnose (Yang et al., 2022b). Nonetheless, there is still a lack of deep and systematic research on the *C. oleifera* anthracnose-resistance mechanisms at the molecular level. Relatively, we still know little about the information of WRKY members in *C. oleifera*. A systematic investigation of CoWRKYs is needed.

Here, we identified WRKY TFs from *C. oleifera* genome, and analysis of their sequence features, conserved motifs, chromosome location, evolutionary relationship, and gene duplication events. Furthermore, the expression patterns of *CoWRKYs* after infection with *C. fructicola* between anthracnose-susceptible and -resistant *C. oleifera* cultivars were also determined. We found *CoWRKY78* showed high expression in leaf and peel, and the expression level of *CoWRKY78* in the anthracnose-resistant *C. oleifera* cultivars showed the greatest decline after inoculation. To investigate its function in anthracnose resistance, we overexpression of *CoWRKY78* in tobacco, and further analysis of physiological changes and the difference in the expression of stress-related genes in WT and *CoWRKY78*-overexpressing lines after inoculation with *Collettrichum nicotianae*. Our study provides valuable guiding information for a deeper investigation of the functional properties and anthracnose defense mechanisms of *C. oleifera* WRKYs.

## 2 Materials and methods

### 2.1 Identification and phylogenetic analysis of WRKYs in *C. oleifera*

Genomic data and annotation information were downloaded from https://github.com/Hengfu-Yin/CON_genome_data. All the WRKY amino acid sequences of *A. thaliana* and *Populus trichocarpa* were obtained from Phytozome ver11 (https://phytozome-next.jgi.doe.gov/). The HMM profile of the WRKY domain was obtained from the Pfam database (PF03106) (El-Gebali et al., 2019), and then used to explore potential WRKYs in *C. oleifera*. We confirm the authenticity of the obtained WRKY sequences by the CDD and SMART (Marchler-Bauer et al., 2017; Letunic and Bork, 2018). According to their positions in the chromosomes of *C. oleifera*, we named these *CoWRKY* genes.

Protein properties of CoWRKY were determined using the online software ProtParam (https://web.expasy.org/protparam/). Furthermore, signal peptide was predicted using SignalP (http://www.cbs.dtu.dk/services/SignalP/) (Duvaud et al., 2021). Subcellular localization was predicted using Plant-mPLoc (http://www.csbio.sjtu.edu.cn/bioinf/plant-multi/) and experiments were performed according to our previous research (Li et al., 2022). Alignment was performed by using the program MUSCLE. Maximum likelihood phylogenetic trees were constructed using the MEGA 7 software with the JTT+G model, and then was visualized using iTOL (Letunic and Bork, 2019).

### 2.2 Analysis of conserved motifs, gene structure, and *cis*-acting elements

We performed conserved motif analysis using MEME version 5.5.0 (parameters: -nmotifs 10 -minw 6 -maxw 50) (Bailey et al., 2009), and gene structures were constructed *via* TBtools software (Chen et al., 2020). Moreover, the PlantCARE was performed to predict and analyze the promoter elements of all *C. oleifera WRKY* genes (Liu et al., 2022). Subsequently, the predicted *cis*-acting elements were visualized using TBtools.

### 2.3 Chromosomal localization and gene duplication events analysis

Based on the *C. oleifera* genome database, the chromosomal locations of *CoWRKYs* were physically mapped on the 15 chromosomes of *C. oleifera*. To analyze the duplication events of *WRKY* genes, MCScanX was run with default parameters except -s (the number of colinear genes to claim a syntenic block) set to 5. Non-synonymous (Ka) and synonymous (Ks) substitutions of identified gene pairs were also calculated using TBtools (Zhao et al., 2020). The synteny of *WRKYs* between *C. oleifera* and the other two species (*A. thaliana* and *P. trichocarpa*) were determined by using Dual Systeny Plotter software and visualized *via* TBtools software.

### 2.4 Plant materials and treatments

Two *C. oleifera* cultivars, MY53 (anthracnose-resistant cultivar) and MY01 (anthracnose-susceptible cultivar) were grown at the *C. oleifera* orchard in Yuping Dong Autonomous County, China (N27°17′, E108°54′). The annual mean temperature and precipitation were 16.4°C (61.52°F) and 1174.1 mm (Wu et al., 2022). These two cultivars had similar genetic backgrounds, but different resistance to anthracnose.

The pathogenic *C. fructicola* was cultured on PDA medium under the dark condition at 28 °C for 1 week. *C. oleifera* young fruits were inoculation with *C. fructicola*. A sterile needle was used to puncture the peels (four wounds per fruit), 10 μL of *C. fructicola* zoospores suspension (1×10^6^ zoospores/mL) was applied to each wound. After the inoculation, plastic tents were constructed to cover the plants and maintain almost 100% humidity by an automatic sprinkler system that switched on every 1 h to facilitate spore germination and infection. The fruits were harvested at 0, 2, 4, and 6 days after inoculation (**Figure S1**) and the peel of the fruits were used for the experiment.

### 2.5 Analysis of *WRKY* gene expression patterns

Using the transcriptome data obtained in our laboratory (NCBI accession number: PRJNA898339), we examined the expression patterns of *C. oleifera WRKYs* after inoculation with *C. fructicola*. A heatmap of *CoWRKYs* was generated by TBtools and the gene expression was estimated by FPKM value (Li et al., 2019). Tissues from the root, stem, leaf, and peel were collected from *C. oleifera* to analyze the tissue-specific expression. As previously described, RNA isolation, cDNA synthesis followed by quantitative real-time RT-PCR (qRT-PCR) analysis were conducted (Wu et al., 2022). Gene expression level was determined *via* the 2^−ΔΔCt^ method, and *EF*1α and *GAPDH* were used as the housekeeping gene. The sequences of all primers are listed in **Table S1**.

### 2.6 Generation of *CoWRKY78*-overexpressing tobacco plants

The coding sequence of *CoWRKY78* was cloned by PCR with primers containing *Bam*H I and *Sac* I restriction enzyme sites. The amplification products were ligated into the pBI121 vector. The recombinant plasmid was introduced into the *Agrobacterium* strain GV3101 and then transformed into the tobacco plants (*N. tabacum* L.cv. NC89) as previously described (Xiong et al., 2020). Firstly, kanamycin-resistant seedlings were analyzed by PCR. We examined the expression level of *CoWRKY78* in transgenic tobacco plants *via* qRT-PCR. The tobacco *actin* and *L25* were used for the normalization of the qRT-PCR analysis.

### 2.7 Assays of resistance of transgenic tobacco against *Collettrichum nicotianae*

The *C. nicotimiae* strain was grown and maintained on the PDA media at 28 °C in the dark for 2 weeks before sporangia collection. The suspension was then incubated at 4°C for 1 h to stimulate the release of zoospores whose concentration was adjusted to 1×10^6^ zoospores/mL. Five-leaf stage seedlings of *CoWRKY78*-overexpressing and WT tobacco plants were treated with *C. nicotimiae* by spraying a zoospores suspension. Inoculated tobacco plants were placed under dark and high humidity conditions for 1 day, then moved to a culture room at 28°C with 16-h/8-h photoperiod cycle. The inoculated leaves were collected for physiological parameter monitoring at 7 days after infection. The lesion areas were quantified *via* ImageJ software.

Based on the lesion area, disease grades were categorized: grade 0, no symptoms; grade 1, few lesions (less than 5%) shown on leaves; grade 2, about 6-10% of leaves area are infected; grade 3, about 11-20% of leaves area are infected; grade 4, about 21-40% of leaves area are infected; grade 5, larger than 41% of leaves area are infected. Disease index (DI) was calculated by the formula: DI (%) = [Σ (rating number × number of plants in the rating)/(the highest rating × total number of plants)] × 100%. Each experiment was performed at least thrice.

### 2.8 Histochemical assays and physiological parameters measurements

Cell death was determined using a trypan blue staining assay (Li et al., 2015). H_2_O_2_ or ${\text{O}}_{2}^{-}$ -accumulation was detected by 3, 3′-diaminobenzidine (DAB) or nitroblue tetrazolium (NBT) staining (Xiong et al., 2020). The malondialdehyde (MDA) content and the activities of superoxide dismutase (SOD), peroxidase (POD) as well as phenylalanine ammonia-lyase (PAL) were measured by the previously reported method (Li et al., 2015).

### 2.9 Analysis of the expression levels and promoter sequences of stress-related genes

QRT-PCR was used to quantify the expression of *NtSOD*, *NtPOD*, *NtPAL*, *NtNPR1*, *NtPR1*, and *NtPDF1.2*. Total RNA was isolated from the leaves of *CoWRKY78*-overexpressing and WT tobacco plants before and after inoculation with *C. nicotianae* and then was converted into cDNA for qRT-PCR analysis. PLACE database was employed to identify W-box in their promoter regions.

### 2.10 Statistical analysis

Results were shown as means ± standard deviations (SD) from three independent experiments replicates. Statistically significant differences (*p*-values below 0.05) were determined *via* one-way ANOVA followed by Tukey’s multiple-comparison test.

## 3 Results

### 3.1 Identification of WRKYs in *C. oleifera*

In the *C. oleifera* genome, 91 C*. oleifera WRKY* genes were identified, and then renamed according to their chromosome distribution (**Table S2**). The longest CoWRKY is CoWRKY42, containing 758 amino acids, while the shortest CoWRKY63 has 133 amino acids (**Table S2**). Moreover, the molecular weights and isoelectric points of CoWRKYs range from 15.04 kDa (CoWRKY63) to 81.99 kDa (CoWRKY42) and 5.01 (CoWRKY86) to 10.17 (CoWRKY50), respectively. The grand average of hydropathicity of the CoWRKYs ranged from -1.216 (CoWRKY70) to -0.391 (CoWRKY17), suggesting that they are all hydrophilic proteins. Furthermore, we found that all CoWRKYs were localized in the nucleus and showed no signal peptide (**Table S2**).

**Figure 1:**
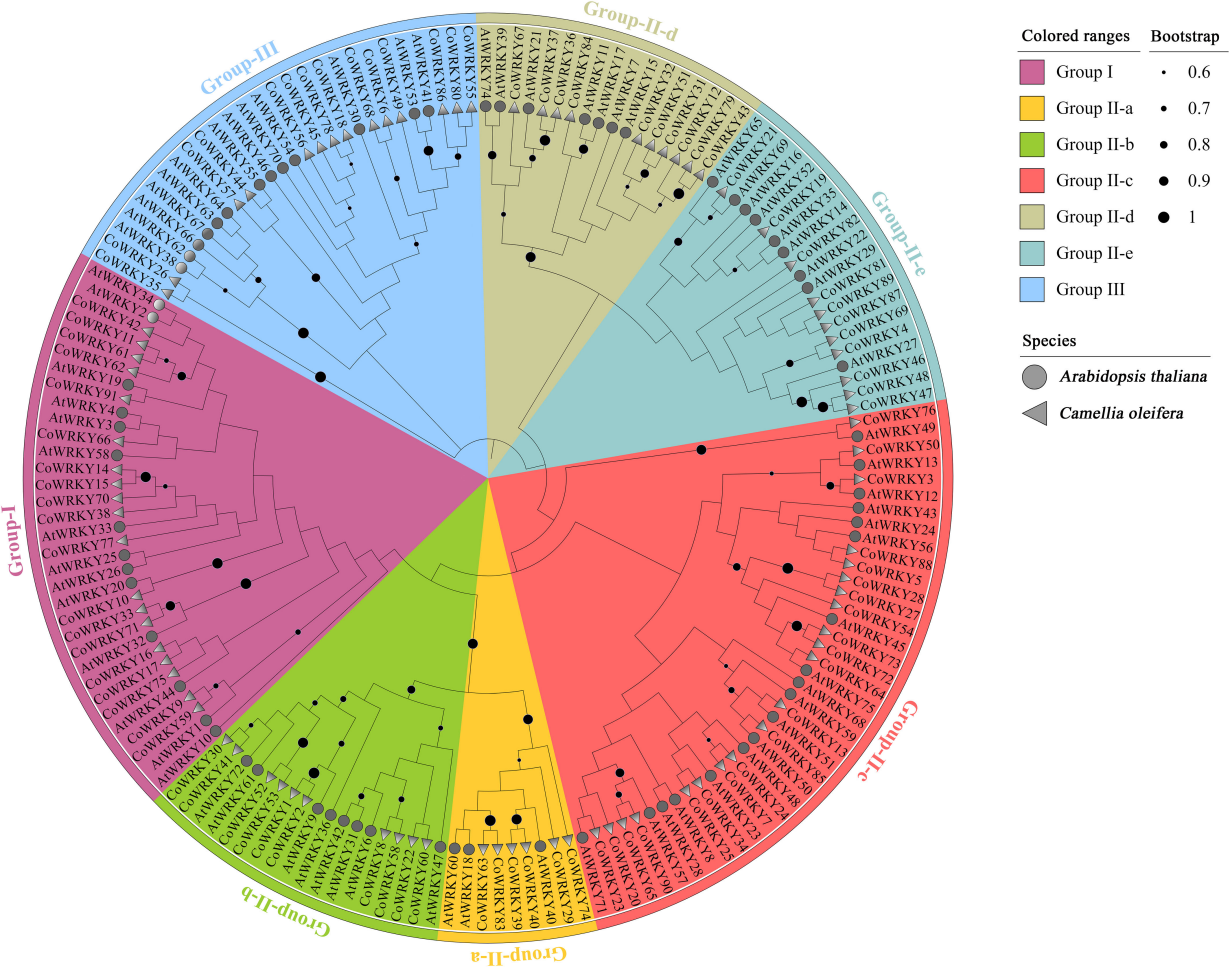
Phylogenetic tree of WRKY TFs from *C. oleifera* and *A. thaliana*. The clades representing different groups were filled with different colors.

We also investigate the evolutionary relationships of WRKYs in *C. oleifera* and *A. thaliana*. Ultimately, 91 CoWRKYs were assigned to 3 groups (**Figure 1**). Group I contained 19 CoWRKY members. According to the phylogenetic tree, group II can be divided into 5 subgroups, 6, 10, 21, 10, and 11 CoWRKY members belonged to the respective group. Moreover, 14 CoWRKY members were assigned to group III.

### 3.2 Structure analysis of the CoWRKYs

**Figure 2:**
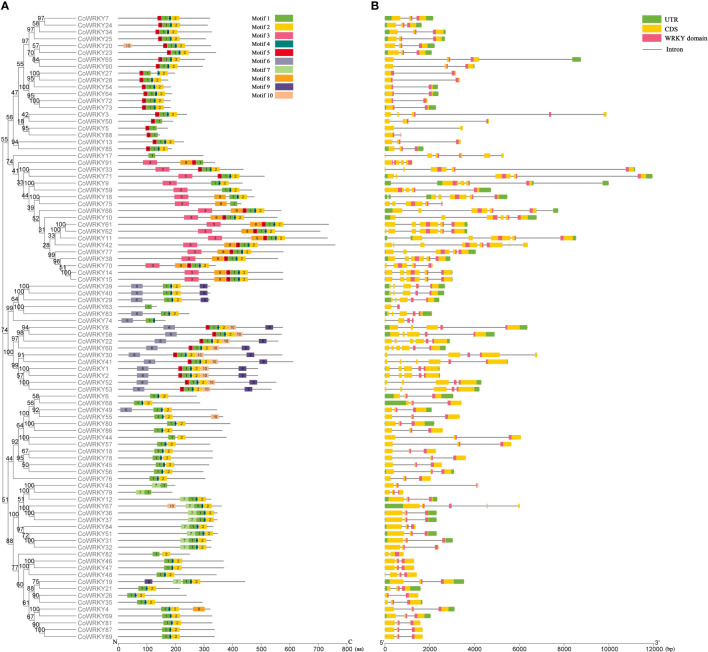
Conserved motifs and gene structure of CoWRKYs in *C. oleifera*. **(A)** Conserved motifs analysis of CoWRKYs. The conserved motifs were displayed in different colors. **(B)** Gene structures analysis of *CoWRKY*s. Different domains were shown in different colors.

The analysis of conserved motifs of *C. oleifera* WRKYs showed that there were 10 conserved motifs in 91 CoWRKYs (**Figure S2**), and the length of these motifs varied from 8 to 50 amino acids (**Table S3**). CoWRKYs in the same group had similar motif compositions (**Figure 2A**). Among the 10 identified motifs, motifs 1 and 3, characterized as WRKY domains. Meanwhile, all CoWRKY members in *C. oleifera* possessed motif 1 (**Figure 2A**). Regarding the gene structure of the *CoWRKYs*, the intron number of the 91 *CoWRKYs* ranged from 1 to 8. *CoWRKY42* had the greatest number of introns (8) (**Figure 2B**).

The *cis*-elements of each *CoWRKY* gene in the 2 kb promoter region were identified using PlantCARE. Three stress response elements were widely distributed in these gene promoters (**Figure S3**). Meanwhile, a variety of plant hormone response elements were also found in their promoters. The results suggested that *C. oleifera* WRKY family may plays an important role in stress and hormone pathways.

### 3.3 Chromosomal locations and gene duplication of *CoWRKYs*

**Figure 3:**
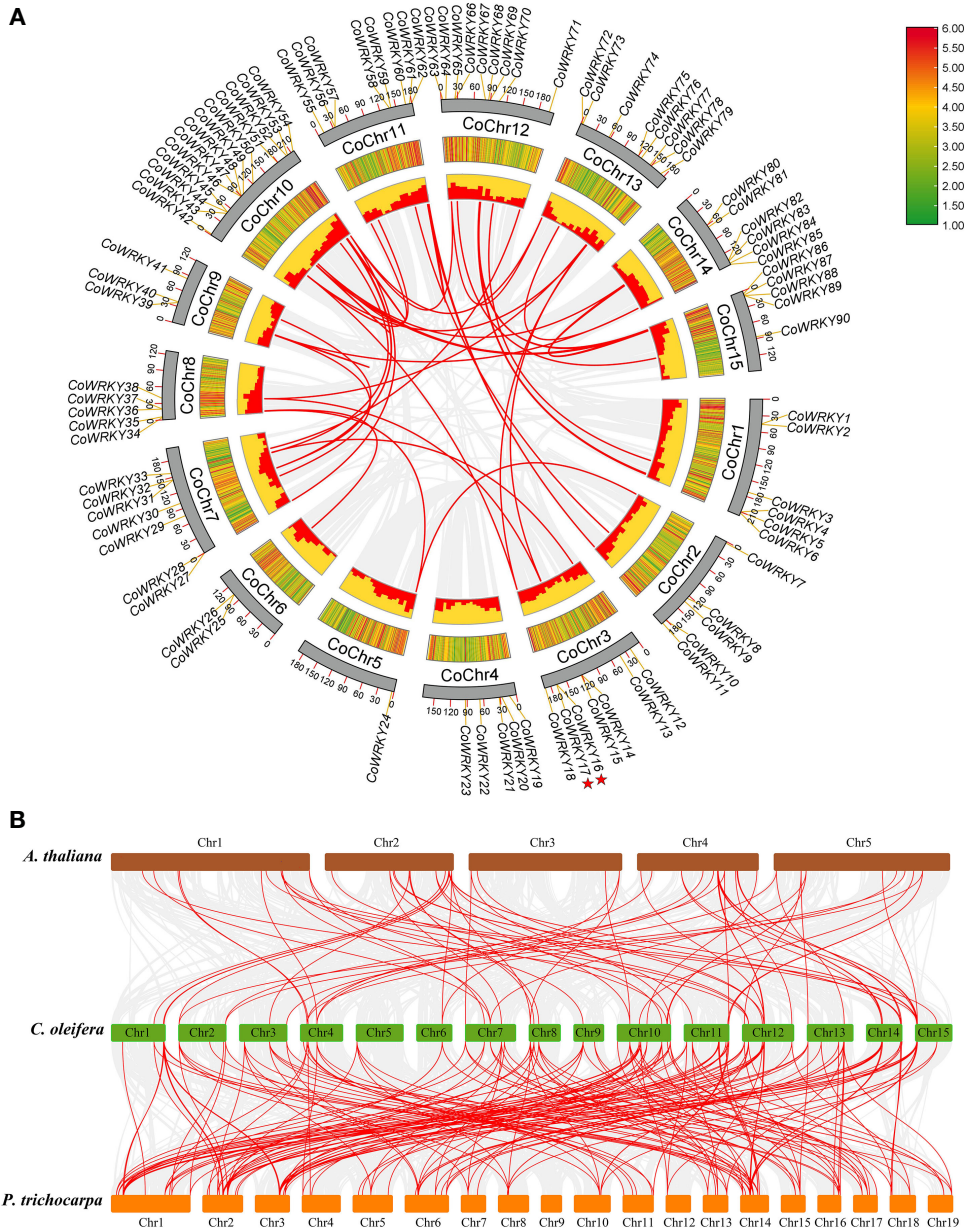
Chromosomal distribution and synteny analysis of *CoWRKYs* in *C*. *oleifera*. **(A)** A total of 90 *WRKY* genes are located in 15 chromosomes. Tandem duplication genes are marked with red stars. Gene pairs of WGD/segmental duplication in *C. oleifera* are linked using red lines. **(B)** Synteny relationship of *WRKY* genes from *A*. *thaliana*, *P. trichocarpa*, and *C. oleifera* genomes. The red lines highlight gene pairs with a collinear relationship. Gray lines in the background indicate the collinear blocks within two genomes.

There are 90 of the 91 *CoWRKY* genes are unevenly distributed on the 15 C*. oleifera* chromosomes (**Figure 3A**). Among these genes, 13 *CoWRKY* genes distributed on chromosome 10, followed by chromosome 12, which had 9 *CoWRKY* genes (**Figure S4**). Chromosomes 11 and 13 had 8 *CoWRKY* genes, and chromosomes 3 and 7 had 7 *CoWRKY* genes. Two chromosomes (chromosome 1 and 14) contained 6 *CoWRKY* genes each, and four chromosomes (chromosome 2, 4, 8, and 15) harbored 5 *CoWRKY* genes each. In addition, 3 *CoWRKY* genes on chromosome 9, 2 *CoWRKY* genes on chromosome 6, and 1 *CoWRKY* gene on chromosome 5.

In this study, we investigated gene duplication events in *A. thaliana*, *P. trichocarpa* and *C. oleifera* genomes. The numbers of whole-genome duplications (WGD)/segmental, tandem, proximal and dispersed duplication events in *A. thaliana* were 30 (41.7%), 2 (2.8%), 2 (2.8%) and 38 (52.7%), respectively (**Table S4**). In *P. trichocarpa*, 90 (92.8%) of the *WRKY* genes originated from WGD/segmental, which indicates WGD/segmental duplication made a valuable contribution to the evolution of the PtWRKY family (**Table S4**). Of the genes of the *C. oleifera* WRKY family, 57 (62.6%) originated from WGD/segmental duplication, 2 (2.2%) appeared to have been created through tandem duplication, 6 (6.6%) were proximal duplicated genes and 26 (28.6%) were dispersed duplicated genes (**Table S4**). These results indicated that WGD/segmental duplication explained the majority of gene duplication events in the CoWRKY family.

We further studied the collinear relationship between *C. oleifera* and two dicotyledons (*A. thaliana* and *P. trichocarpa*) (**Figure 3B**). The 74 orthologous gene pairs were identified between *C. oleifera* and *A. thaliana* (**Table S5**). In comparison to *C. oleifera* and *P. trichocarpa* genomes, 196 gene pairs were observed (**Table S6**). Significantly, among these gene pairs, 48 C*. oleifera WRKYs* have collinear relationships with *A. thaliana* and *P. trichocarpa*. Nine *C. oleifera WRKY* genes, including *CoWRKY 7, 20, 22, 34, 54, 68, 69, 80*, and *86*, were associated with at least 6 syntenic gene pairs, indicating that they might have played a crucial role in *C. oleifera* WRKYs evolution. Moreover, the synonymous substitution rates (Ka/Ks) of the gene pairs were calculated to identify the evolutionary forces. All of the 243 orthologous gene pairs had Ka/Ks< 1, suggesting that purifying selection may be the dominant force driving the evolution of *CoWRKY* genes.

### 3.4 Analysis of expression patterns of *CoWRKYs* in response to *C. fructicola* infection

**Figure 4:**
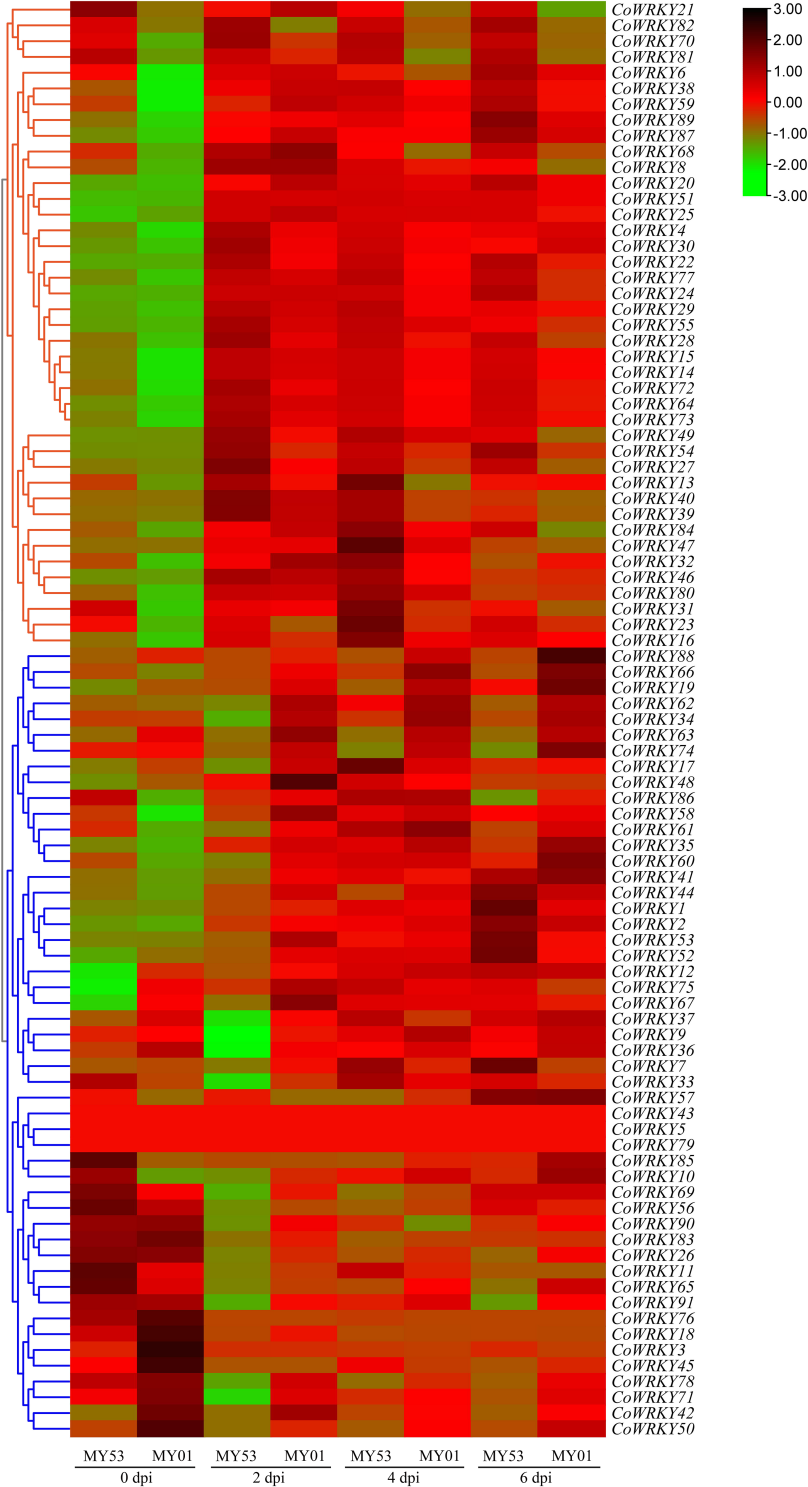
Heatmap of the expression profiles of *CoWRKY* genes responsive to *C. fructicola*.

To screen the potential *CoWRKY*s in response to anthracnose infection, we created a heatmap to compare the profile of expression of *CoWRKY* genes in peels of two *C. oleifera* cultivars after *C. fructicola* inoculation. In group I, the *CoWRKY* genes in the anthracnose-resistant cultivars of *C. oleifera* have a higher expression level than that in the anthracnose-susceptible cultivars of *C. oleifera* after inoculation with *C. fructicola*. In another group, the expression level of *CoWRKY*s in the susceptible cultivar occurred earlier and stronger while being induced later in the resistant cultivar (**Figure 4**).

We selected 6 *CoWRKY* members from 2 groups for qRT-PCR analysis (**Figure S5**). *CoWRKY4*, *CoWRKY28*, and *CoWRKY82* were from group I, which showed higher expression level in the anthracnose-resistant *C. oleifera* cultivars than that in the anthracnose-susceptible *C. oleifera* cultivars after inoculation with *C. fructicola*. In contrast, after inoculation, *CoWRKY36*, *CoWRKY74*, and *CoWRKY78* in group II had lower expression levels in the anthracnose-resistant *C. oleifera* cultivars than in the anthracnose-susceptible *C. oleifera* cultivars (**Figure S5**). These expression patterns were consistent with the results from transcriptome sequencing. It is noteworthy that, *CoWRKY78* in the resistant cultivar showed the greatest decline in expression level at 2 d after inoculation. Furthermore, *CoWRKY78* showed high expression levels in the leaf and peel (**Figure S6A**). Therefore, *CoWRKY78* was selected to explore its function in response to anthracnose infection.

### 3.5 Anthracnose resistance analysis of *CoWRKY78*-overexpressing tobacco plants

Under confocal microscope, the green fluorescence signal was observed mainly in the nucleus of tobacco leaf epidermal cells, suggesting that CoWRKY78 is located in the nucleus (**Figure S6B**). To investigate *CoWRKY78* function in anthracnose resistance, the pBI121-*CoWRKY78* overexpression construct was transformed into tobacco. Firstly, kanamycin-resistant seedlings were verified by PCR. The result showed that the transgenic lines (lines 1, 2, 3, 5, and 6) exhibited expected bands (**Figure S7A**). We further verified the expression level of *CoWRKY78* using qRT-PCR analysis. The line 1 showed 3.1-fold higher expression, while the line 5 showed a 2.87-fold higher level relative to line 6. Hence, these lines were selected for further experiments (**Figure S7B**).

**Figure 5:**
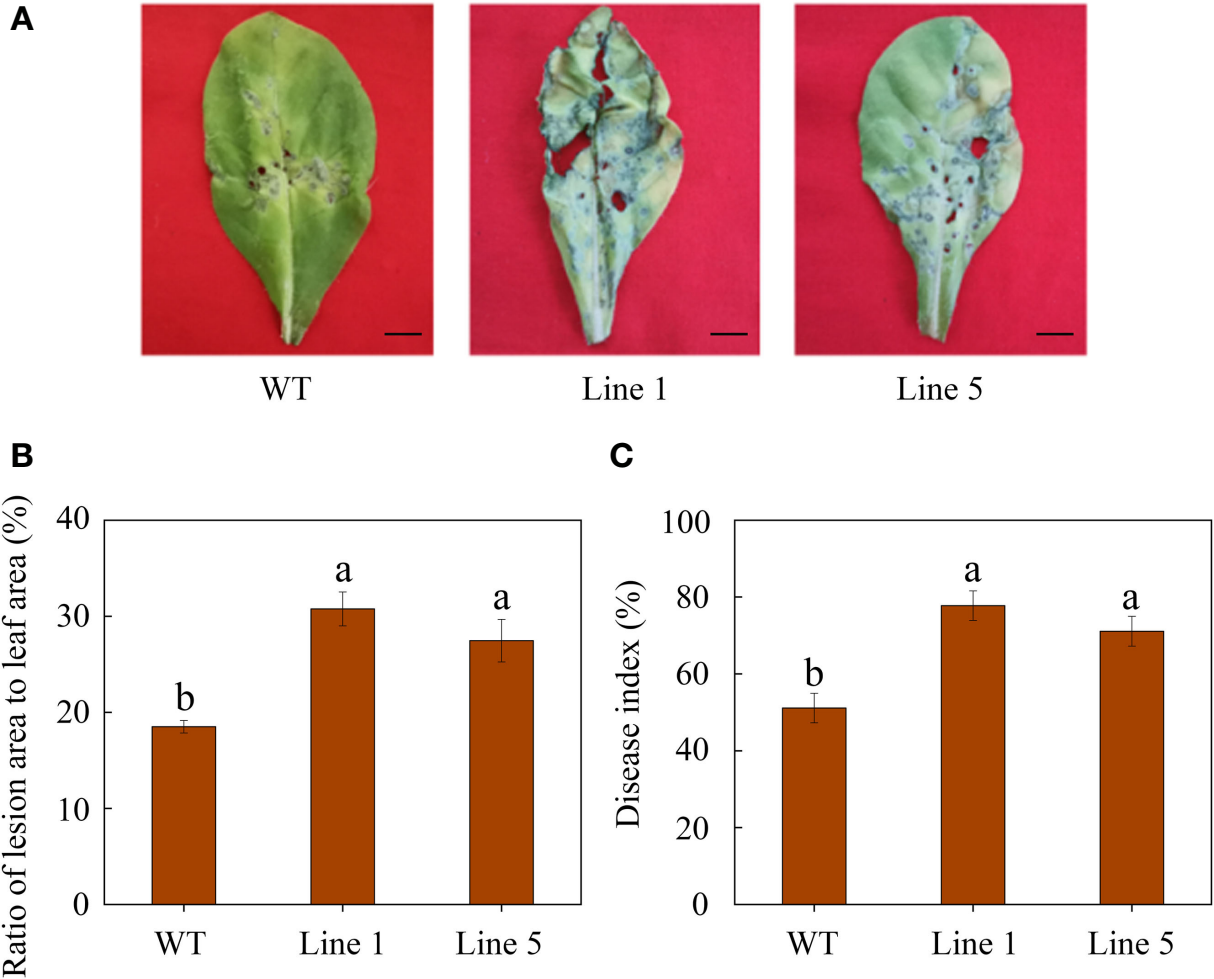
Overexpression of *CoWRKY78* decreased anthracnose resistance in transgenic tobacco. **(A)** Disease symptoms on the leaves from transgenic and WT plants at 7 days after *C. nicotianae* inoculation. Scale bar indicates 1 cm. **(B)** The ratio of lesion area to leaf area in inoculated tobacco leaves. **(C)** Measurement of DI at 7 days after *C. nicotianae* inoculation. Data represent the means ± SD and bars denoted by a different letter are significantly different (*p*-values below 0.05).

**Figure 6:**
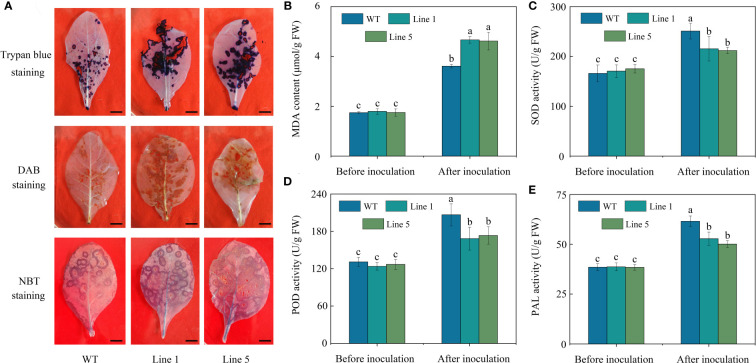
Physiological changes in transgenic and WT tobacco plants before and after *C. nicotianae* inoculation. **(A)** Histochemical staining *via* trypan blue, DAB, and NBT. Scale bar indicates 1 cm. **(B)** MDA content. **(C)** SOD activity. **(D)** POD activity. **(E)** PAL activity. Data represent the means ± SD and bars denoted by a different letter are significantly different (*p*-values below 0.05).

After inoculation, *CoWRKY78*-overexpressing tobacco plants developed more severe disease symptoms on the leaves compared with WT tobacco plants (**Figure 5A**). Moreover, transgenic plants showed a higher ratio of lesion area to whole leaf area than WT tobacco plants after inoculation (**Figure 5B**). Furthermore, *CoWRKY78*-overexpressing tobacco plants had higher DI than WT plants (**Figure 5C**), suggesting that the areas of anthracnose lesions were more prominent in transgenic tobacco plants. After inoculation, we found that cell death was more prominent in the *CoWRKY78*-overexpressing tobacco plants than in the WT tobacco plants according to trypan blue staining (**Figure 6A**, upper panel). These results suggested that overexpression of *CoWRKY78* led to decreased resistance to anthracnose in tobacco.

ROS and MDA often accumulate in plants after pathogen infection (Li et al., 2015). After *C. nicotianae* inoculation, more intense brown coloration (**Figure 6A**, middle panel), blue coloration (**Figure 6A**, lower panel), and higher MDA content (**Figure 6B**) in the transgenic tobacco plants than that in WT plants. Antioxidant enzymes like SOD or POD play an important role in removing excess ROS. PAL activity is often used as one of the important indicators for plant resistance evaluation (Li et al., 2015). The activities of SOD (**Figure 6C**), POD (**Figure 6D**), and PAL (**Figure 6E**) in the transgenic tobacco plants were significantly lower compared with those in the WT plants after inoculation with *C. nicotianae*. These data suggested that overexpression of *CoWRKY78* in tobacco resulted in decreased activities of defense-related enzymes, which lead to increased sensitivity to anthracnose.

### 3.6 Expression analysis of stress-related genes

**Figure 7:**
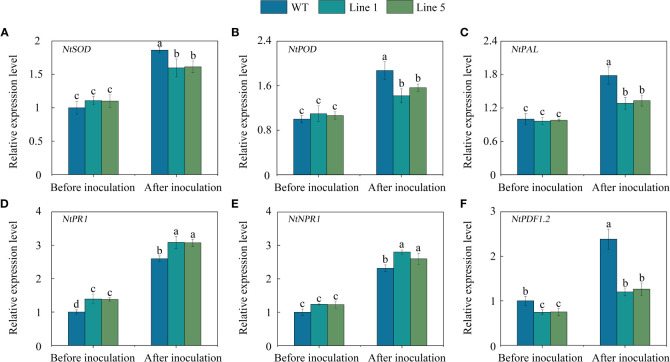
The expression of some stress-related genes in the transgenic and WT tobacco plants. **(A-F)** Analysis of expression of *NtSOD*, *NtPOD*, *NtPAL*, *NtPR1*, *NtNPR1*, and *NtPDF1.2* in the leaves of transgenic and WT tobacco plants before and after *C. nicotianae* inoculation. Data represent the means ± SD and bars denoted by a different letter are significantly different (*p*-values below 0.05).

To gain further insight into the regulated role of CoWRKY78, we further analysed the expression profiles of several stress-related genes which are related to ROS-scavenging (*NtSOD* and *NtPOD*), pathogen challenge (*NtPAL*), and pathogen defense (*NtPR1*, *NtNPR1*, and *NtPDF1.2*). No significant differences were observed in the expression of *NtSOD* (**Figure 7A**), *NtPOD* (**Figure 7B**), *NtPAL* (**Figure 7C**), and *NtNPR1* (**Figure 7E**) between *CoWRKY78*-overexpressing and WT tobacco plants before inoculation. Interestingly, the transgenic lines exhibited higher expression of *NtPR1* (**Figure 7D**), but lower expression of *NtPDF1.2* (**Figure 7F**) than the WT plants. After inoculation, all of these genes were upregulated. Compared with WT tobacco plants, the expression levels of *NtSOD*, *NtPOD*, *NtPAL*, and *NtPDF1.2* were lower in transgenic tobacco plants, but the expression of *NtNPR1* and *NtPR1* was significantly higher in transgenic plants. Furthermore, we observed several W-box in their promoter regions (**Table S7**), suggesting CoWRKY78 may participate in anthracnose resistance by regulating these genes expression.

## 4 Discussion

### 4.1 The evolution of *C. oleifera* WRKYs

WRKY TFs have been reported in many woody plants, including 104 WRKYs in poplar (He et al., 2012), 80 WRKYs in grape (Zhang and Feng, 2014), 132 WRKYs in *Musa balbisiana* (Goel et al., 2016), 56 WRKYs in tea (Wang et al., 2019), and 103 WRKYs in common walnut (Hao et al., 2021). As an important woody oil plant, not much is known about the exact number of WRKYs in *C. oleifera*, so we began our investigations.

We identified 91 WRKY TFs in *C. oleifera*, which can be subdivided into 3 groups (**Figure 1**). There were 19, 58, and 14 members that were included in group I, II, and III, respectively. Understanding the exon and intron organization of these genes may help us to obtain more information about their evolutionary history (Liu et al., 2022). Regarding the structure of WRKY family in *C. oleifera*, the intron number of the 91 *CoWRKYs* ranged from 1 to 8 (**Figure 2**). Obvious differences in gene structures exist in *CoWRKY*s, but members clustered in the same group exhibited similar structures, implying the important roles of these features have led to functional divergence.

The duplication of genes plays an important role in gene family evolution (Yan et al., 2022). Previous research has established that the three WGD in *A. thaliana* have been directly responsible for over 90% of the increase in TFs, signal transducers, and developmental genes in the last 350 million years (Maere et al., 2005). We investigated gene duplication events in *C. oleifera* genomes, 57 (62.6%) of the WRKY genes originated from WGD/segmental duplication, which indicates WGD/segmental duplication made a valuable contribution to the evolution of the *C. oleifera* WRKY family. Our finding that is aligns with the previous findings (Waqas et al., 2019; Yan et al., 2022). Moreover, orthologous relationships of *WRKY* genes among *C. oleifera*, *A. thaliana*, and *P. trichocarpa* genomes were detected, including *C. oleifera*-*A. thaliana* (74 pairs) and *C. oleifera*-*P. trichocarpa* (196 pairs) (**Figure 3B**). The synonymous substitution rates (Ka/Ks) of the gene pairs were calculated to identify the evolutionary forces. All of the 243 orthologous gene pairs had Ka/Ks< 1, suggesting that purifying selection may be the dominant force driving the evolution of *CoWRKY* genes.

### 4.2 The roles of WRKY TF family members in *C. oleifera*

Anthracnose, caused by *C. fructicola*, is an extremely destructive disease of *C. oleifera* (Zhang et al., 2019b). The pathogen attacks many *C. oleifera* organs, including buds, fruits, and leaves. WRKY TFs are of great importance in plant-pathogen interactions. However, there have not been any reports investigating the expression pattern of *WRKY* genes directly involved in *C. oleifera*-*C. fructicola* interaction. A systematic investigation of CoWRKYs is needed.

In this study, the peels of two *C. oleifera* cultivars showing different resistance to anthracnose were analyzed by transcriptomics. We created a heatmap to analyze the profile of expression of *CoWRKY* genes in peels of two *C. oleifera* cultivars after *C. fructicola* inoculation. In group I, the expression level of *CoWRKY*s in the resistant *C. oleifera* cultivar occurred stronger and earlier, while *CoWRKY* genes in group II have a lower expression level in the anthracnose-resistant *C. oleifera* cultivars than that in the anthracnose- susceptible *C. oleifera* cultivars (**Figure 4**). Our analysis provides a unique opportunity to understand the candidate *WRKY* genes involved in the anthracnose resistance.

A majority of the *WRKY* genes have been shown to respond to stress and phytohormone treatments (Xiong et al., 2020; Lim et al., 2022). Consistent with previous results, we found that abundant *cis*-acting regulatory elements in *CoWRKY* promoters were related to abiotic stress (drought and cold) and hormones (ABA, MeJA, and SA). A total of 33 (35.2%) *CoWRKY* genes have MBS, implying their important roles in drought stress. In addition, 70 (76.9%) *CoWRKY* genes have ABA-responsive element, implying they also plays a critical role in ABA signaling pathways. With ecological value, *C. oleifera* can grow well in drought and barren soil (He et al., 2022b). Our finding show that *CoWRKY* genes were important and necessary for the responses to drought stress and further exploration of the potential biological functions of *CoWRKY* genes is needed.

### 4.3 CoWRKY78-altered anthracnose resistance is potentially related to ROS homeostasis

*CoWRKY78* showed high expression in leaf and peel, and its expression in the resistant cultivar showed the greatest decline at 2 d after inoculation. Therefore, *CoWRKY78* was selected to explore its function in response to anthracnose infection. Overexpression of *CoWRKY78* decreased anthracnose resistance in transgenic tobacco plants, which was demonstrated by a higher ratio of lesion area to leaf area, higher DI, and more severe cell death than WT plants (**Figures 5**, **6A**).

Pathogen infection can cause oxidative stress by increasing the production of ROS in plants (Bloem et al., 2015; Li et al., 2015). However, late massive ROS generations may lead to damage to cellular membranes. MDA, SOD, and POD activities are common indicators for assessing plant resistance to diseases (Li et al., 2015; Prabhukarthikeyan et al., 2018). Previous studies showed that the accumulation of MDA, SOD, and POD in both disease-resistant and susceptible *C. oleifera* lines increased by anthracnose infection. However, disease resistant lines exhibited lower MDA, but higher SOD and POD activities compared to susceptible lines (Yang et al., 2022b). To investigate the physiological differences between WT and *CoWRKY78*-overexpression tobacco plants before and after anthracnose infection, these important physiological indices were measured. After inoculation, the transgenic tobacco plants accumulated more ROS and MDA than WT plants (**Figures 6A, B**). Moreover, the activities of SOD and POD in the transgenic tobacco plants were lower than those in the WT plants (**Figures 6C, D**). Furthermore, overexpression of *CoWRKY78* decreased the expression of *NtSOD* and *NtPOD* (**Figures 7A, B**), which was consistent with the enzyme activity. These suggested that CoWRKY78 negatively regulates the resistance of anthracnose *via* impairing the antioxidant abilities, which in turn excess accumulation of ROS.

## 5 Conclusion

We identified 91 WRKY TFs in *C. oleifera*, which can be divided into 3 groups. Segmental duplications were the main contributor to the expansion of *C. oleifera* WRKY TF family. We mined multiple anthracnose-responsive *CoWRKY* genes. Overexpression of *CoWRKY78* in tobacco resulted in increased sensitivity to anthracnose. The transgenic tobacco plants had higher ROS and lower activity of defense-related enzymes than WT tobacco plants. Furthermore, the expression of stress-related genes involved in ROS-homeostasis was also reduced in the *CoWRKY78*-overexpressing plants. These findings increase our understanding of the *C. oleifera* WRKYs and provide further evidence for exploration of anthracnose resistance mechanisms in *C. oleifera*.

## Data availability statement

The datasets presented in this study can be found in online repositories. The names of the repository/repositories and accession number(s) can be found below: https://www.ncbi.nlm.nih.gov/, PRJNA898339.

## Author contributions

JL and CR conceived and designed the research. JL and CX conducted the bioinformatics analysis and performed the experiments. DR, WD, and HL analyzed data. JL wrote the manuscript. All authors contributed to the article and approved the submitted version.

## Supplementary material

Supplementary Figure 1 Phenotype of MY53 and MY01 after inoculation with *C. fructicola*. Scale bar indicates 1 cm.

Supplementary Figure 2 Sequences of conserved motifs.

Supplementary Figure 3 The *cis*-acting regulatory elements in the promoters of *C. oleifera WRKY* genes.

Supplementary Figure 4 Chromosome distribution of *C. oleifera WRKY* genes.

Supplementary Figure 5 Expression analysis of selected *CoWRKY* genes using qRT-PCR. (A-F) The expression profiles of 6 C*. oleifera WRKY* genes after inoculation with *C. fructicola* in MY53 and MY01 peels. Data represent the means ± SD and bars denoted by a different letter are significantly different (*p*-values below 0.05).

Supplementary Figure 6 Characterization of CoWRKY78. **(A)** Tissue-specific expression patterns analysis of *CoWRKY78*. Data represent the means ± SD and bars denoted by a different letter are significantly different (*p*-values below 0.05). **(B)** Subcellular localization of CoWRKY78. Scale bar indicates 20 μm in all images.

Supplementary Figure 7 Molecular analysis of putatively transgenic tobacco plants. **(A)** Genomic DNA-PCR analysis of transgenic plants. H: blank control (The amplified PCR products using the H_2_O as templates); P: positive control (The amplified PCR products using the pBI121-*CoWRKY78* plasmid DNA as templates); M: DL2000 DNA Marker. **(B)** QRT-PCR analysis of the selected transgenic lines for determination of the expression level of *CoWRKY78*. Data represent the means ± SD and bars denoted by a different letter are significantly different (*p*-values below 0.05).
