# Supplementary figures and images for: Identification of Camellia oleifera WRKY transcription factor genes and functional characterization of CoWRKY78
Source: Front Plant Sci. 2023 Mar 9;14:1110366. doi: 10.3389/fpls.2023.1110366 (PMC10036053; doi:10.3389/fpls.2023.1110366)

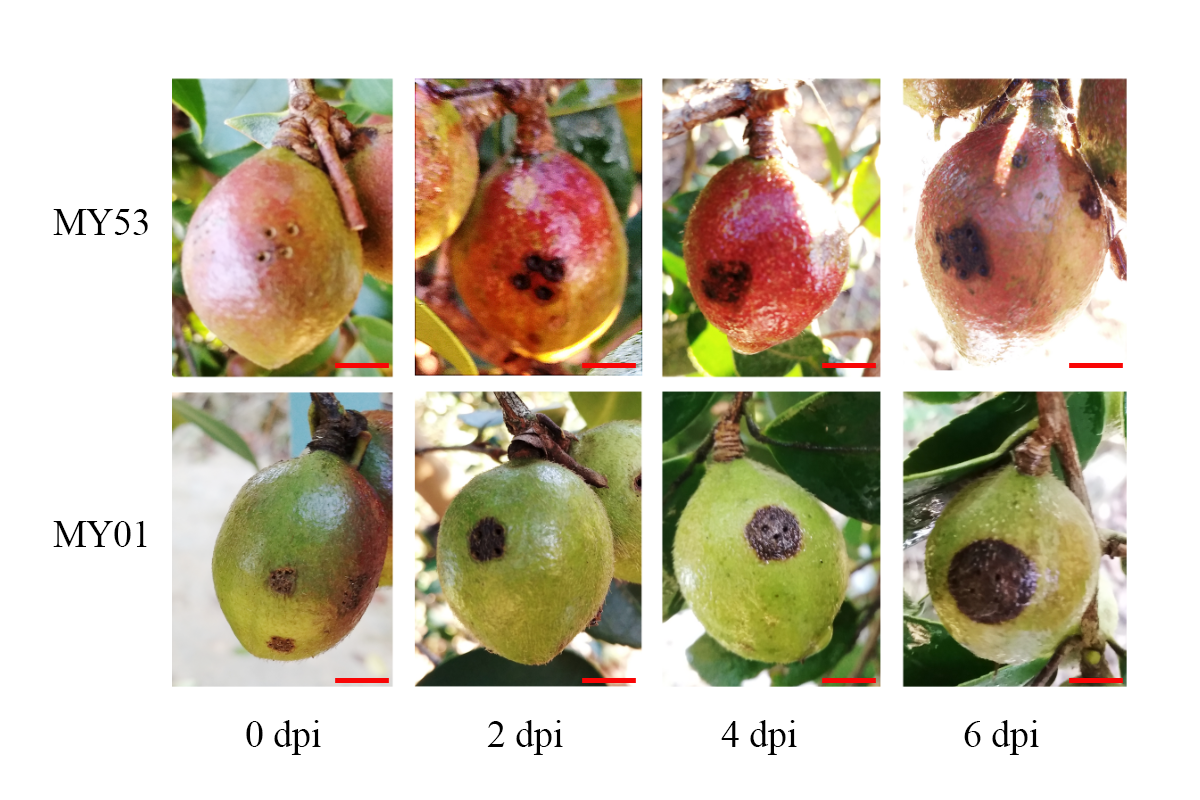

Supplement: Supplementary Figure 1 — Phenotype of MY53 and MY01 after inoculation with C. fructicola. Scale bar indicates 1 cm. [file Image_1.tif]

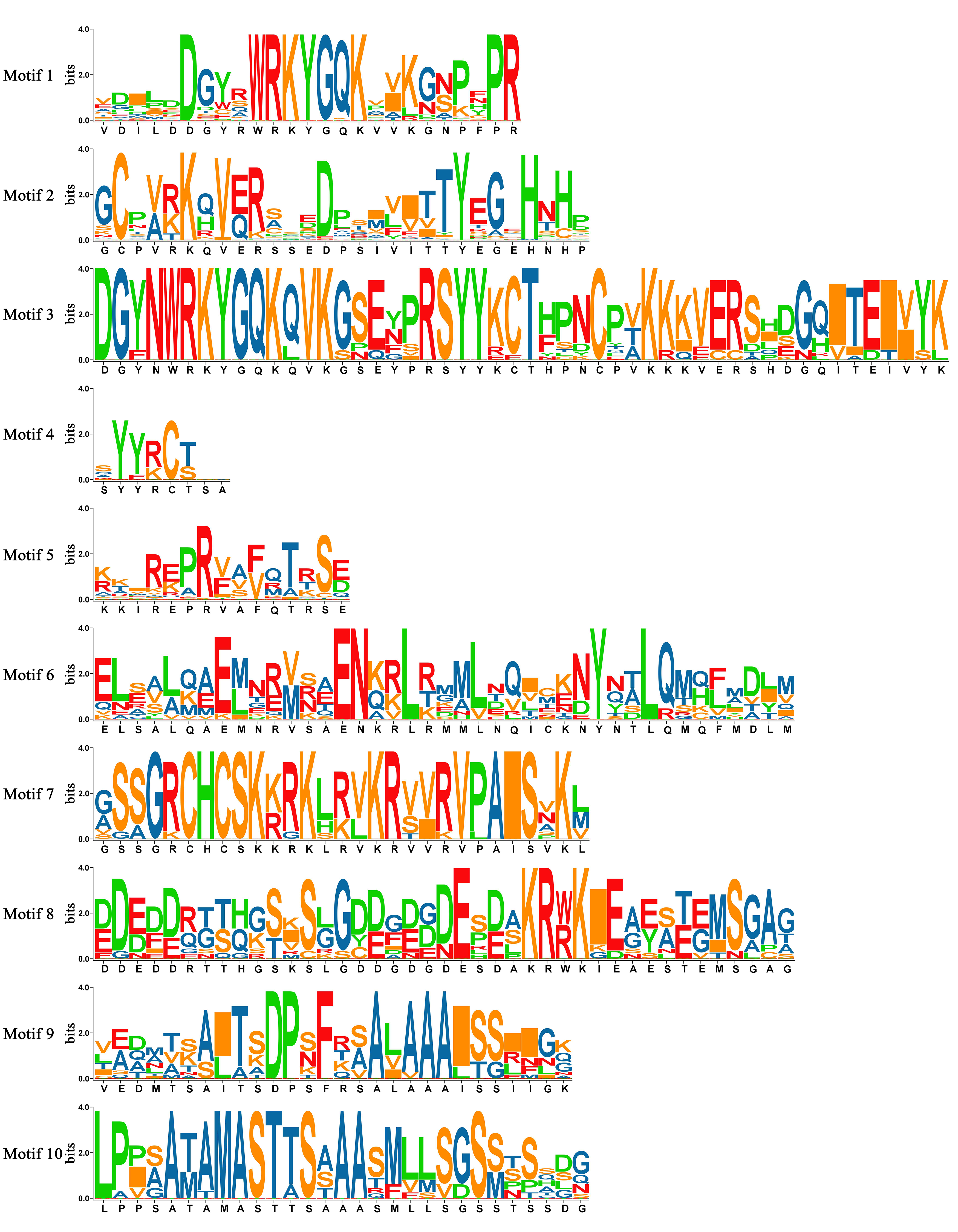

Supplement: Supplementary Figure 2 — Sequences of conserved motifs. [file Image_2.tif]

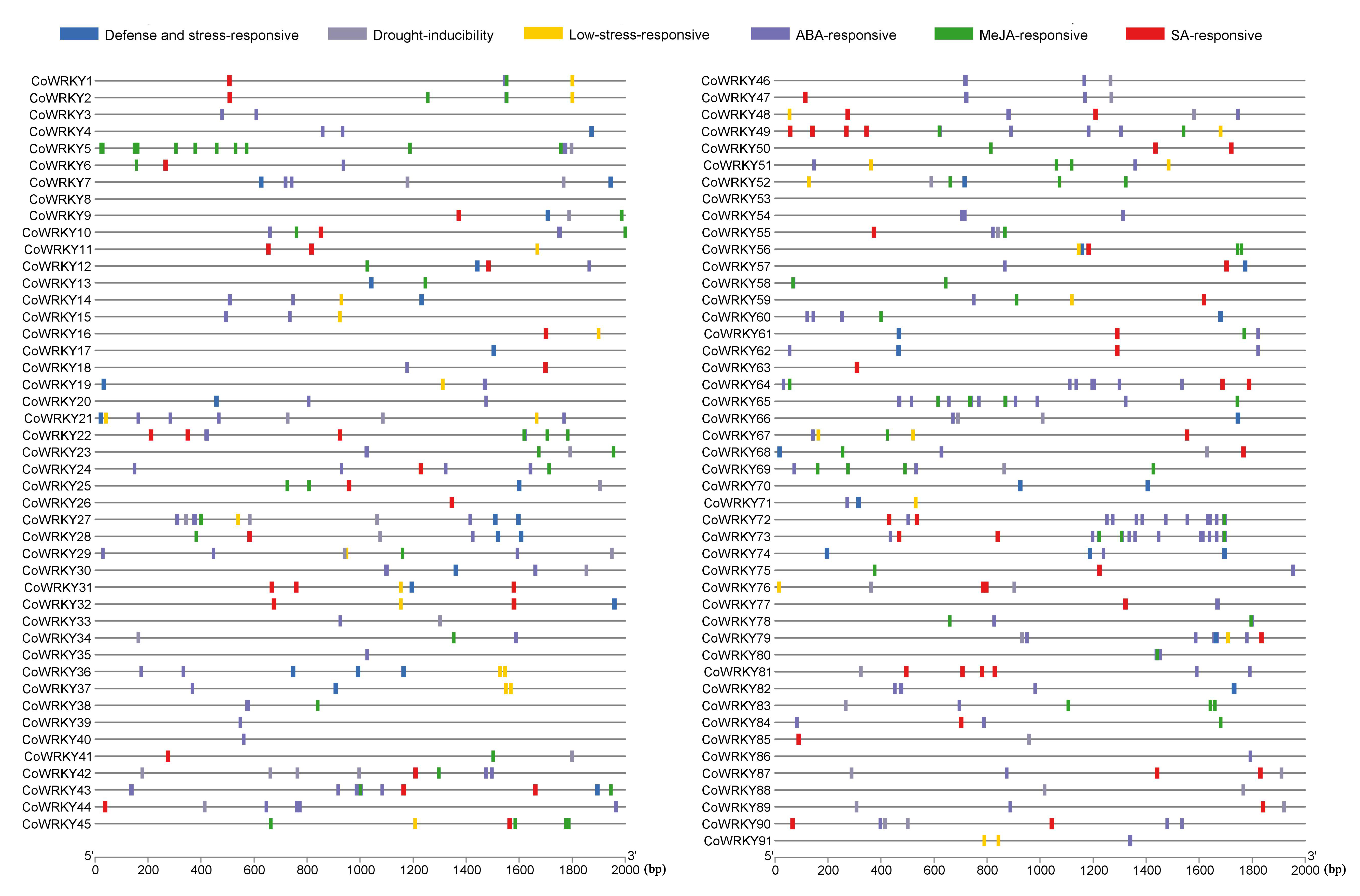

Supplement: Supplementary Figure 3 — The cis-acting regulatory elements in the promoters of C. oleifera WRKY genes. [file Image_3.tif]

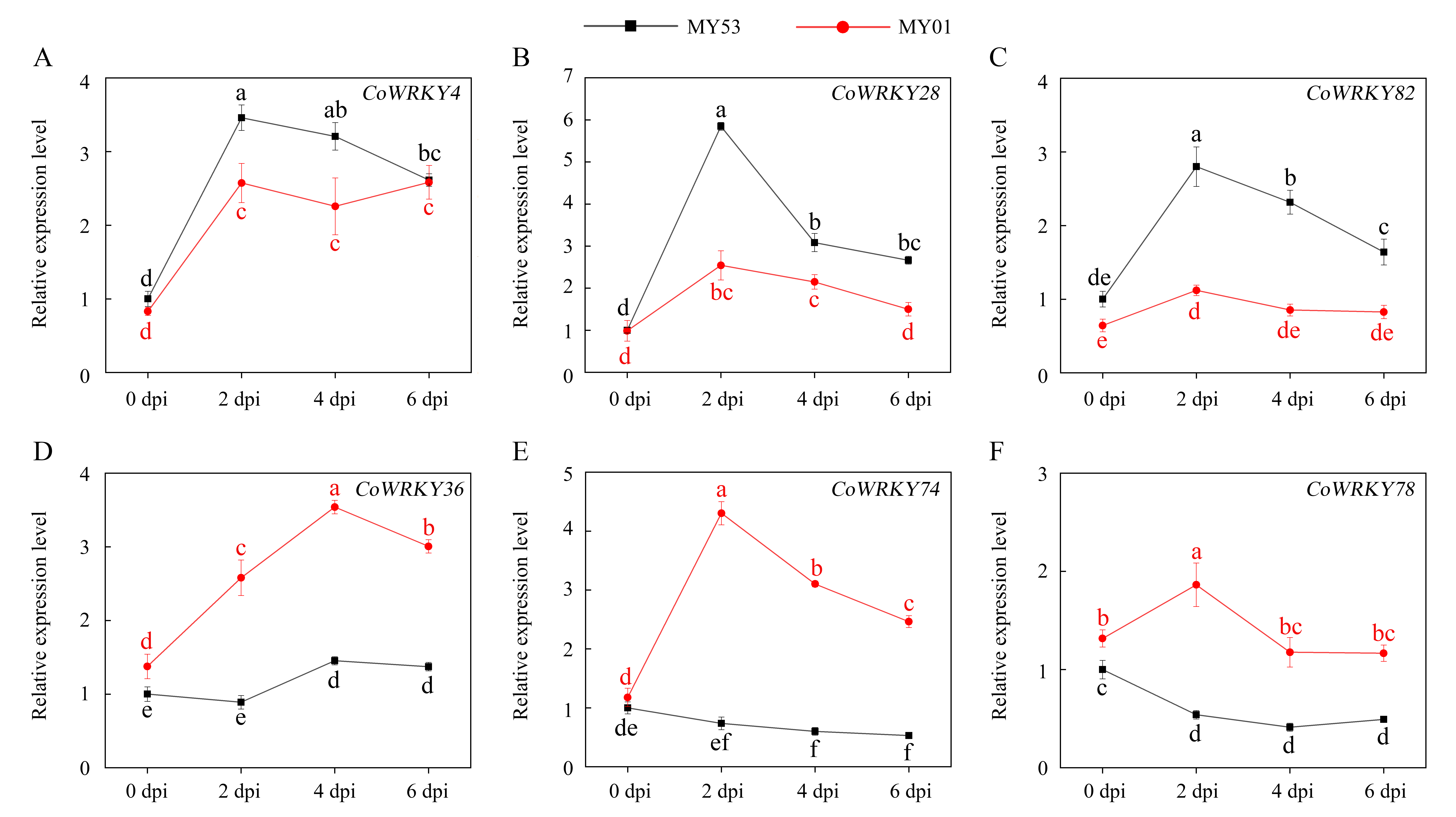

Supplement: Supplementary Figure 5 — Expression analysis of selected CoWRKY genes using qRT-PCR. (A-F) The expression profiles of 6 C. oleifera WRKY genes after inoculation with C. fructicola in MY53 and MY01 peels. Data represent the means ± SD and bars denoted by a different letter are significantly different (p-values below 0.05). [file Image_5.tif]

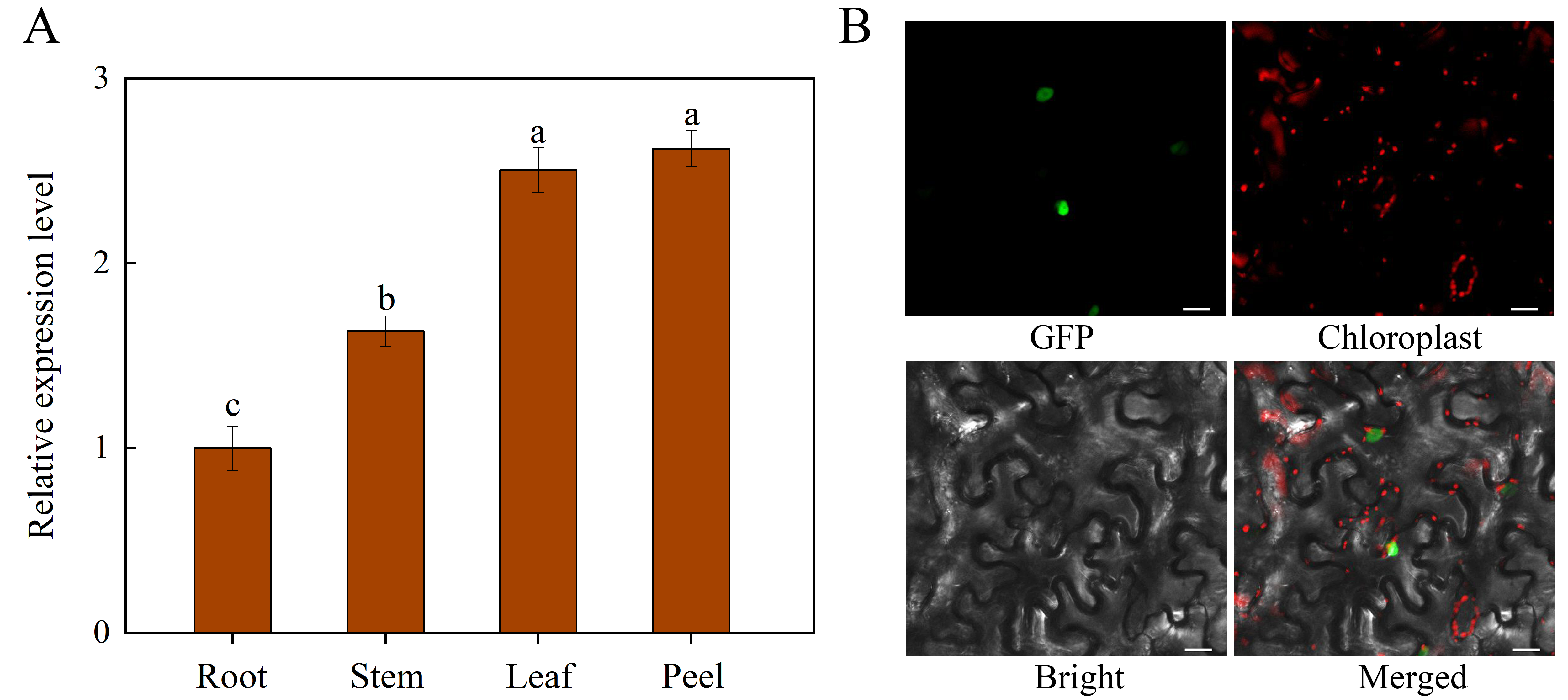

Supplement: Supplementary Figure 6 — Characterization of CoWRKY78. (A) Tissue-specific expression patterns analysis of CoWRKY78. Data represent the means ± SD and bars denoted by a different letter are significantly different (p-values below 0.05). (B) Subcellular localization of CoWRKY78. Scale bar indicates 20 μm in all images. [file Image_6.tif]

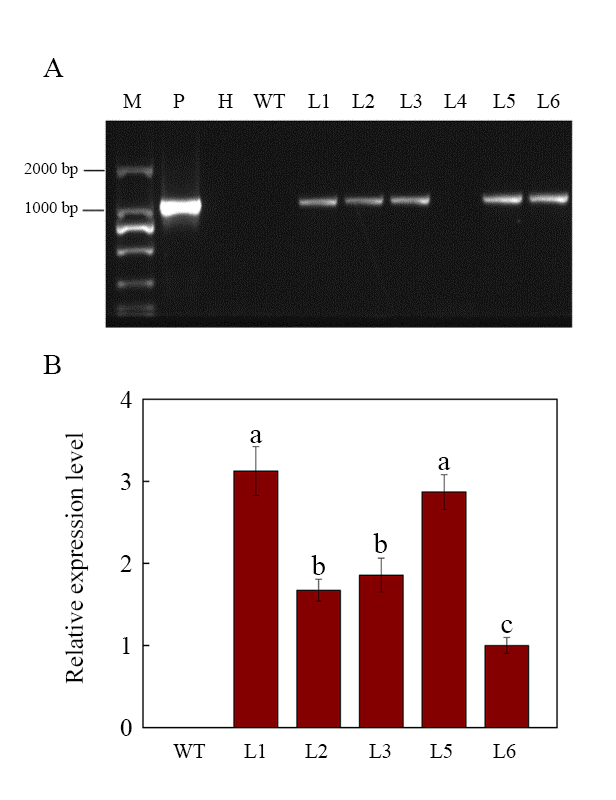

Supplement: Supplementary Figure 7 — Molecular analysis of putatively transgenic tobacco plants. (A) Genomic DNA-PCR analysis of transgenic plants. H: blank control (The amplified PCR products using the H2O as templates); P: positive control (The amplified PCR products using the pBI121-CoWRKY78 plasmid DNA as templates); M: DL2000 DNA Marker. (B) QRT-PCR analysis of the selected transgenic lines for determination of the expression level of CoWRKY78. Data represent the means ± SD and bars denoted by a different letter are significantly different (p-values below 0.05). [file Image_7.tif]
