# Supplementary material for: Identification of Camellia oleifera WRKY transcription factor genes and functional characterization of CoWRKY78
Source: Front Plant Sci. 2023 Mar 9;14:1110366. doi: 10.3389/fpls.2023.1110366 (PMC10036053; doi:10.3389/fpls.2023.1110366)
Supplement: Supplementary file 8 [file Table_1.docx]

**TABLE S1. The primers used in this experiment.**

| Name | Primer sequence (5' to 3') | Description and purpose |
| --- | --- | --- |
| *QCoWRKY4* | CAACAAAGGCAGCATCATCAA | QRT-PCR |
|  | AACCCTCTTCATCTGGTTCTTC |  |
| *QCoWRKY28* | CAACAAGGGTGCAATGTGAAG | QRT-PCR |
|  | GTAGGCTTCTGTACTGGATGTG |  |
| *QCoWRKY82* | GAAACAAGTGCGAAGCAAAGA | QRT-PCR |
|  | GTGTAGTGCAGGAGAGAGTATTG |  |
| *QCoWRKY36* | GTTGCTAATTTGGACGGGAATG | QRT-PCR |
|  | CTTCCAGAGCACCTTCTCTTATG |  |
| *QCoWRKY74* | CACATCCAGAGGAAGAAGGAAG | QRT-PCR |
|  | GGATCTGCGGTCACCATATT |  |
| *QCoWRKY78* | GGTCCTACTCAACTTCGGTTC | QRT-PCR |
|  | GGTAGTGGTGGTTGGGAAATA |  |
| *QCoEF*1α | TCGAGAAGGAGCCCAAATTC | QRT-PCR |
|  | TGGGTACTCCGAGAAAGTCT |  |
| *QCoGAPDH* | GGTGCCAAGAAGGTGGTAATA | QRT-PCR |
|  | GTTGTGCAGCTTGCATTAGAG |  |
| *CoWRKY78* | ATGGACTCATCTTTGCCGGA | Amplify the ORF regions |
|  | TCAAAATTCAAATTGCAAGACGTCGT |  |
| *CoWRKY78-GFP* | CGAGCTCATGGACTCATCTTTGCCGGA | Subcellular localization |
|  | GCTCTAGAAAATTCAAATTGCAAGACGTCGT |  |
| *QNtSOD* | GGTGATTGAAACCACTGCTAATC | QRT-PCR |
|  | GCATGTTCCCAAACGTCTATTC |  |
| *QNtPOD* | CTCCATTTCCATGACTGCTTTG | QRT-PCR |
|  | GTTGGGTGGTGAGGTCTTT |  |
| *QNtPAL* | AGCTAGTAGTGATTGGGTTATGG | QRT-PCR |
|  | CCGTTCTTGGTTCTCCTATGT |  |
| *QNtPR1* | GTTGAGATGTGGGTCGATGAG | QRT-PCR |
|  | CGCCAAACCACCTGAGTATAG |  |
| *QNtNPR1* | CGCTTCTTTCCACGTTGTTC | QRT-PCR |
|  | TTGACGCTCTTCTGCTGTATC |  |
| *QNtPDF1.2* | CTGTACCAGCCCTTGCTAAT | QRT-PCR |
|  | TAGCTAGCACGTCCATCTTTG |  |
| *QNtActin* | TGGATTCTGGTGATGGTGTTAG | QRT-PCR |
|  | GTCAAGTCACGACCAGCTAAA |  |
| *QNtL25* | GCTAAGGTTGCCAAGGCTGTC | QRT-PCR |
|  | TAAGGTATTGACTTTCTTTGTCTGA |  |
