# Supplementary material for: Identification of Camellia oleifera WRKY transcription factor genes and functional characterization of CoWRKY78
Source: Front Plant Sci. 2023 Mar 9;14:1110366. doi: 10.3389/fpls.2023.1110366 (PMC10036053; doi:10.3389/fpls.2023.1110366)
Supplement: Supplementary file 9 [file Table_2.docx]

**TABLE S2.** **The length distribution and physicochemical properties of *C. oleifera* WRKY proteins.**

| Name | Group | ID | Peptide length (aa) | MW (kDa) | Isoelectric point | GRAVY score | Signal peptides | Subcellular localization |
| --- | --- | --- | --- | --- | --- | --- | --- | --- |
| CoWRKY1 | Group II-b | maker-HiC_scaffold_1-snap-gene-450.28-mRNA-1 | 487 | 53.27 | 5.84 | -0.823 | No | Nucleus |
| CoWRKY2 | Group II-b | maker-HiC_scaffold_1-snap-gene-456.25-mRNA-1 | 487 | 53.26 | 5.84 | -0.826 | No | Nucleus |
| CoWRKY3 | Group II-c | maker-HiC_scaffold_1-snap-gene-1716.15-mRNA-1 | 238 | 27.06 | 8.51 | -0.829 | No | Nucleus |
| CoWRKY4 | Group II-e | maker-HiC_scaffold_1-snap-gene-2096.37-mRNA-1 | 320 | 35.43 | 6.12 | -0.827 | No | Nucleus |
| CoWRKY5 | Group II-c | snap_masked-HiC_scaffold_1-processed-gene-2097.9-mRNA-1 | 171 | 19.25 | 9.47 | -0.739 | No | Nucleus |
| CoWRKY6 | Group III | maker-HiC_scaffold_1-snap-gene-2120.24-mRNA-1 | 272 | 30.34 | 5.79 | -0.526 | No | Nucleus |
| CoWRKY7 | Group II-c | maker-HiC_scaffold_2-snap-gene-23.39-mRNA-1 | 319 | 35.57 | 6.83 | -0.866 | No | Nucleus |
| CoWRKY8 | Group II-b | maker-HiC_scaffold_2-snap-gene-1252.34-mRNA-1 | 574 | 62.64 | 5.81 | -0.772 | No | Nucleus |
| CoWRKY9 | Group I | maker-HiC_scaffold_2-snap-gene-1326.0-mRNA-4 | 433 | 48.29 | 8.92 | -1.104 | No | Nucleus |
| CoWRKY10 | Group I | maker-HiC_scaffold_2-snap-gene-1815.40-mRNA-1 | 555 | 60.92 | 6.11 | -0.734 | No | Nucleus |
| CoWRKY11 | Group I | maker-HiC_scaffold_2-snap-gene-1890.40-mRNA-1 | 731 | 79.10 | 6.18 | -0.855 | No | Nucleus |
| CoWRKY12 | Group II-d | maker-HiC_scaffold_3-snap-gene-199.22-mRNA-1 | 323 | 36.04 | 9.61 | -0.808 | No | Nucleus |
| CoWRKY13 | Group II-c | maker-HiC_scaffold_3-snap-gene-422.22-mRNA-1 | 227 | 25.95 | 8.9 | -0.798 | No | Nucleus |
| CoWRKY14 | Group I | genemark-HiC_scaffold_3-processed-gene-1204.28-mRNA-1 | 575 | 63.60 | 7.2 | -0.986 | No | Nucleus |
| CoWRKY15 | Group I | maker-HiC_scaffold_3-snap-gene-1212.25-mRNA-1 | 575 | 63.60 | 7.2 | -0.986 | No | Nucleus |
| CoWRKY16 | Group I | maker-HiC_scaffold_3-snap-gene-1689.2-mRNA-2 | 475 | 52.35 | 8.5 | -0.923 | No | Nucleus |
| CoWRKY17 | Group I | maker-HiC_scaffold_3-snap-gene-1690.6-mRNA-1 | 296 | 32.43 | 9.12 | -0.391 | No | Nucleus |
| CoWRKY18 | Group III | maker-HiC_scaffold_3-snap-gene-1902.40-mRNA-1 | 329 | 37.42 | 6.78 | -0.871 | No | Nucleus |
| CoWRKY19 | Group II-e | maker-HiC_scaffold_4-snap-gene-129.0-mRNA-1 | 442 | 48.19 | 5.17 | -0.686 | No | Nucleus |
| CoWRKY20 | Group II-c | snap_masked-HiC_scaffold_4-processed-gene-268.25-mRNA-1 | 322 | 35.72 | 6.35 | -0.748 | No | Nucleus |
| CoWRKY21 | Group II-e | snap_masked-HiC_scaffold_4-processed-gene-314.32-mRNA-1 | 215 | 24.43 | 8.43 | -0.974 | No | Nucleus |
| CoWRKY22 | Group II-b | maker-HiC_scaffold_4-snap-gene-670.30-mRNA-1 | 558 | 60.49 | 8.43 | -0.604 | No | Nucleus |
| CoWRKY23 | Group II-c | maker-HiC_scaffold_4-snap-gene-929.20-mRNA-1 | 340 | 37.49 | 6.2 | -0.833 | No | Nucleus |
| CoWRKY24 | Group II-c | maker-HiC_scaffold_5-snap-gene-37.29-mRNA-1 | 311 | 34.35 | 6.01 | -0.686 | No | Nucleus |
| CoWRKY25 | Group II-c | maker-HiC_scaffold_6-snap-gene-1030.13-mRNA-1 | 304 | 34.23 | 6.66 | -0.747 | No | Nucleus |
| CoWRKY26 | Group III | maker-HiC_scaffold_6-snap-gene-1034.38-mRNA-1 | 237 | 27.09 | 5.35 | -1.041 | No | Nucleus |
| CoWRKY27 | Group II-c | maker-HiC_scaffold_7-snap-gene-0.19-mRNA-1 | 196 | 22.33 | 9.39 | -0.643 | No | Nucleus |
| CoWRKY28 | Group II-c | maker-HiC_scaffold_7-snap-gene-3.3-mRNA-1 | 173 | 19.78 | 9.56 | -0.769 | No | Nucleus |
| CoWRKY29 | Group II-a | maker-HiC_scaffold_7-snap-gene-762.23-mRNA-1 | 318 | 35.23 | 8.51 | -0.701 | No | Nucleus |
| CoWRKY30 | Group II-b | maker-HiC_scaffold_7-snap-gene-1001.51-mRNA-1 | 572 | 61.81 | 8.36 | -0.707 | No | Nucleus |
| CoWRKY31 | Group II-d | maker-HiC_scaffold_7-snap-gene-1446.56-mRNA-1 | 323 | 35.35 | 9.47 | -0.48 | No | Nucleus |
| CoWRKY32 | Group II-d | maker-HiC_scaffold_7-snap-gene-1452.25-mRNA-1 | 323 | 35.35 | 9.41 | -0.48 | No | Nucleus |
| CoWRKY33 | Group I | maker-HiC_scaffold_7-snap-gene-1568.0-mRNA-1 | 436 | 48.01 | 9.01 | -0.711 | No | Nucleus |
| CoWRKY34 | Group II-c | maker-HiC_scaffold_8-snap-gene-49.28-mRNA-1 | 325 | 36.05 | 8.81 | -0.536 | No | Nucleus |
| CoWRKY35 | Group III | maker-HiC_scaffold_8-snap-gene-55.45-mRNA-1 | 293 | 33.17 | 5.18 | -1.018 | No | Nucleus |
| CoWRKY36 | Group II-d | maker-HiC_scaffold_8-snap-gene-195.13-mRNA-1 | 345 | 38.96 | 9.86 | -0.861 | No | Nucleus |
| CoWRKY37 | Group II-d | maker-HiC_scaffold_8-snap-gene-197.4-mRNA-1 | 345 | 39.07 | 9.94 | -0.85 | No | Nucleus |
| CoWRKY38 | Group I | maker-HiC_scaffold_8-snap-gene-391.28-mRNA-1 | 557 | 61.61 | 8.44 | -0.92 | No | Nucleus |
| CoWRKY39 | Group II-a | snap_masked-HiC_scaffold_9-processed-gene-372.11-mRNA-1 | 320 | 34.98 | 8.81 | -0.661 | No | Nucleus |
| CoWRKY40 | Group II-a | maker-HiC_scaffold_9-snap-gene-385.6-mRNA-1 | 320 | 34.97 | 8.82 | -0.659 | No | Nucleus |
| CoWRKY41 | Group II-b | maker-HiC_scaffold_9-snap-gene-837.0-mRNA-1 | 611 | 66.62 | 7.93 | -0.777 | No | Nucleus |
| CoWRKY42 | Group I | maker-HiC_scaffold_10-snap-gene-19.24-mRNA-1 | 758 | 81.99 | 6.21 | -0.539 | No | Nucleus |
| CoWRKY43 | Group II-d | snap_masked-HiC_scaffold_10-processed-gene-485.3-mRNA-1 | 197 | 22.27 | 10.02 | -0.619 | No | Nucleus |
| CoWRKY44 | Group III | maker-HiC_scaffold_10-snap-gene-512.0-mRNA-1 | 377 | 39.82 | 5.79 | -0.615 | No | Nucleus |
| CoWRKY45 | Group III | maker-HiC_scaffold_10-snap-gene-512.5-mRNA-1 | 316 | 34.86 | 5.1 | -0.539 | No | Nucleus |
| CoWRKY46 | Group II-e | maker-HiC_scaffold_10-snap-gene-885.13-mRNA-1 | 367 | 40.65 | 8.24 | -0.89 | No | Nucleus |
| CoWRKY47 | Group II-e | maker-HiC_scaffold_10-snap-gene-895.1-mRNA-1 | 368 | 40.81 | 6.61 | -0.902 | No | Nucleus |
| CoWRKY48 | Group II-e | maker-HiC_scaffold_10-snap-gene-895.4-mRNA-1 | 342 | 37.96 | 8.26 | -0.989 | No | Nucleus |
| CoWRKY49 | Group III | maker-HiC_scaffold_10-snap-gene-952.46-mRNA-1 | 344 | 38.47 | 5.95 | -0.652 | No | Nucleus |
| CoWRKY50 | Group II-c | maker-HiC_scaffold_10-snap-gene-1201.18-mRNA-1 | 190 | 21.20 | 10.17 | -0.726 | No | Nucleus |
| CoWRKY51 | Group II-d | maker-HiC_scaffold_10-snap-gene-1797.44-mRNA-1 | 346 | 38.25 | 9.65 | -0.607 | No | Nucleus |
| CoWRKY52 | Group II-b | maker-HiC_scaffold_10-snap-gene-2000.0-mRNA-1 | 551 | 60.72 | 6 | -0.818 | No | Nucleus |
| CoWRKY53 | Group II-b | maker-HiC_scaffold_10-snap-gene-2023.14-mRNA-1 | 534 | 58.96 | 5.99 | -0.813 | No | Nucleus |
| CoWRKY54 | Group II-c | genemark-HiC_scaffold_10-processed-gene-2141.55-mRNA-1 | 182 | 20.80 | 9.45 | -0.845 | No | Nucleus |
| CoWRKY55 | Group III | maker-HiC_scaffold_11-snap-gene-109.19-mRNA-1 | 365 | 40.04 | 5.22 | -0.808 | No | Nucleus |
| CoWRKY56 | Group III | maker-HiC_scaffold_11-snap-gene-334.20-mRNA-1 | 296 | 33.36 | 5.09 | -0.568 | No | Nucleus |
| CoWRKY57 | Group III | maker-HiC_scaffold_11-snap-gene-335.3-mRNA-1 | 319 | 35.10 | 6.45 | -0.579 | No | Nucleus |
| CoWRKY58 | Group II-b | maker-HiC_scaffold_11-snap-gene-1383.0-mRNA-1 | 570 | 63.27 | 6.75 | -0.801 | No | Nucleus |
| CoWRKY59 | Group I | maker-HiC_scaffold_11-snap-gene-1414.2-mRNA-1 | 466 | 51.48 | 6.15 | -1.085 | No | Nucleus |
| CoWRKY60 | Group II-b | maker-HiC_scaffold_11-snap-gene-1686.2-mRNA-1 | 462 | 51.12 | 7.58 | -0.617 | No | Nucleus |
| CoWRKY61 | Group I | snap_masked-HiC_scaffold_11-processed-gene-1762.0-mRNA-1 | 735 | 79.56 | 5.96 | -0.767 | No | Nucleus |
| CoWRKY62 | Group I | snap_masked-HiC_scaffold_11-processed-gene-1799.0-mRNA-1 | 705 | 76.32 | 6.21 | -0.806 | No | Nucleus |
| CoWRKY63 | Group II-a | maker-HiC_scaffold_12-snap-gene-31.45-mRNA-1 | 133 | 15.04 | 9.3 | -1.02 | No | Nucleus |
| CoWRKY64 | Group II-c | maker-HiC_scaffold_12-snap-gene-85.32-mRNA-1 | 186 | 21.05 | 9.28 | -0.83 | No | Nucleus |
| CoWRKY65 | Group II-c | maker-HiC_scaffold_12-snap-gene-247.26-mRNA-1 | 301 | 33.39 | 5.5 | -0.919 | No | Nucleus |
| CoWRKY66 | Group I | snap_masked-HiC_scaffold_12-processed-gene-251.13-mRNA-1 | 569 | 61.97 | 7.69 | -0.806 | No | Nucleus |
| CoWRKY67 | Group II-d | snap_masked-HiC_scaffold_12-processed-gene-808.22-mRNA-1 | 360 | 40.52 | 9.69 | -0.714 | No | Nucleus |
| CoWRKY68 | Group III | maker-HiC_scaffold_12-snap-gene-865.29-mRNA-1 | 284 | 32.26 | 5.02 | -0.701 | No | Nucleus |
| CoWRKY69 | Group II-e | maker-HiC_scaffold_12-snap-gene-890.38-mRNA-1 | 326 | 35.64 | 5.92 | -0.779 | No | Nucleus |
| CoWRKY70 | Group I | maker-HiC_scaffold_12-snap-gene-1055.4-mRNA-1 | 339 | 38.66 | 8.48 | -1.216 | No | Nucleus |
| CoWRKY71 | Group I | maker-HiC_scaffold_12-snap-gene-1993.11-mRNA-1 | 510 | 55.78 | 5.69 | -0.855 | No | Nucleus |
| CoWRKY72 | Group II-c | maker-HiC_scaffold_13-snap-gene-31.13-mRNA-1 | 181 | 20.50 | 9.4 | -0.824 | No | Nucleus |
| CoWRKY73 | Group II-c | maker-HiC_scaffold_13-snap-gene-36.1-mRNA-1 | 181 | 20.53 | 9.4 | -0.826 | No | Nucleus |
| CoWRKY74 | Group II-a | maker-HiC_scaffold_13-snap-gene-568.29-mRNA-1 | 163 | 18.71 | 9.6 | -0.645 | No | Nucleus |
| CoWRKY75 | Group I | maker-HiC_scaffold_13-snap-gene-1207.8-mRNA-1 | 429 | 46.92 | 8.59 | -0.873 | No | Nucleus |
| CoWRKY76 | Group II-c | maker-HiC_scaffold_13-snap-gene-1268.20-mRNA-1 | 303 | 33.79 | 5.05 | -0.651 | No | Nucleus |
| CoWRKY77 | Group I | maker-HiC_scaffold_13-snap-gene-1470.11-mRNA-1 | 576 | 64.02 | 8.09 | -1.001 | No | Nucleus |
| CoWRKY78 | Group III | maker-HiC_scaffold_13-snap-gene-1490.5-mRNA-1 | 329 | 36.49 | 6.05 | -0.716 | No | Nucleus |
| CoWRKY79 | Group II-d | maker-HiC_scaffold_13-snap-gene-1721.18-mRNA-1 | 187 | 20.80 | 9.45 | -0.587 | No | Nucleus |
| CoWRKY80 | Group III | genemark-HiC_scaffold_14-processed-gene-612.11-mRNA-1 | 390 | 44.28 | 5.99 | -0.721 | No | Nucleus |
| CoWRKY81 | Group II-e | genemark-HiC_scaffold_14-processed-gene-646.22-mRNA-1 | 327 | 37.07 | 5.4 | -0.689 | No | Nucleus |
| CoWRKY82 | Group II-e | maker-HiC_scaffold_14-snap-gene-1354.0-mRNA-1 | 248 | 28.19 | 6.61 | -0.999 | No | Nucleus |
| CoWRKY83 | Group II-a | maker-HiC_scaffold_14-snap-gene-1369.27-mRNA-1 | 247 | 27.79 | 6.99 | -0.847 | No | Nucleus |
| CoWRKY84 | Group II-d | maker-HiC_scaffold_14-snap-gene-1409.1-mRNA-1 | 330 | 35.98 | 9.7 | -0.63 | No | Nucleus |
| CoWRKY85 | Group II-c | maker-HiC_scaffold_14-snap-gene-1433.3-mRNA-1 | 185 | 20.57 | 5.76 | -0.917 | No | Nucleus |
| CoWRKY86 | Group III | maker-HiC_scaffold_15-snap-gene-54.0-mRNA-1 | 362 | 40.73 | 5.01 | -0.759 | No | Nucleus |
| CoWRKY87 | Group II-e | maker-HiC_scaffold_15-snap-gene-77.17-mRNA-1 | 335 | 36.61 | 5.14 | -0.589 | No | Nucleus |
| CoWRKY88 | Group II-c | maker-HiC_scaffold_15-snap-gene-84.27-mRNA-1 | 144 | 15.84 | 9.41 | -0.818 | No | Nucleus |
| CoWRKY89 | Group II-e | maker-HiC_scaffold_15-snap-gene-84.28-mRNA-1 | 335 | 36.62 | 5.24 | -0.604 | No | Nucleus |
| CoWRKY90 | Group II-c | maker-HiC_scaffold_15-snap-gene-868.5-mRNA-1 | 294 | 32.14 | 5.93 | -0.86 | No | Nucleus |
| CoWRKY91 | Group I | maker-scaffold_290_fragment_25-snap-gene-0.8-mRNA-1 | 336 | 36.93 | 6.11 | -1.041 | No | Nucleus |
