# Supplementary material for: Identification of Camellia oleifera WRKY transcription factor genes and functional characterization of CoWRKY78
Source: Front Plant Sci. 2023 Mar 9;14:1110366. doi: 10.3389/fpls.2023.1110366 (PMC10036053; doi:10.3389/fpls.2023.1110366)
Supplement: Supplementary file 10 [file Table_3.docx]

**TABLE S3. Summary of motifs in WRKY TFs by MEME analysis.**

| Motif | Length | Number | Sequence | Annotation |
| --- | --- | --- | --- | --- |
| 1 | 25 | 90 | VDILDDGY[RS]WRKYGQK[PV][VI]K[GN][NS]P[FHN]PR | WRKY domain |
| 2 | 29 | 81 | GCP[VA][RK]K[QH]V[EQ]R[SA]S[ED]DP[ST][IM][VL][IV][TV]TYEGEH[NT]HP | Low complexity region |
| 3 | 50 | 16 | DGYNWRKYGQKQVKGSE[YN]PRSYYKCT[HF]PNCP[VTA]KKKVERS[HI]DG[QH]ITEI[VI]YK | WRKY domain |
| 4 | 8 | 81 | SYY[RK]C[TS]SA | No match |
| 5 | 15 | 39 | [KR][KT][IV]R[EK]PR[VF][AVS][FV][QMR]T[RTK]S[ED] | Low complexity region |
| 6 | 41 | 14 | EL[ES][AVS][LA][QKM][AE]E[ML]N[RE][MV][NRS][AE]EN[KQ][RK]L[RTK][MG][MA]L[NDT][QV][ILV][CM][KE][ND]Y[NQY][AT]LQM[HQ][FL][MA]D[LIY][MV] | Coil |
| 7 | 29 | 10 | [GA]S[SA]GRCHCSK[KR]RK[LH][RK][VL]KR[VS][VI]RVPAIS[NVA]K[LMV] | Zn-cluster domain |
| 8 | 41 | 10 | [DE]D[ED][DF][DE][RQ][GT][TS][HQ][GK][ST][IKV]S[GL]G[DCY][DE][GF][DE][DGE][DN]E[SP][DE][ASL]KR[RW]KIE[AG][EY][AS][ET][EG][MI]S[GA]A[GAT] | No match |
| 9 | 28 | 12 | [VL][AE][DAQ][AM][TAV][KS][AS][IL]T[SK]DP[SN]F[RKT][AS]A[LV]AAAI[ST][SG][ILR]IG[KGQ] | Low complexity region |
| 10 | 29 | 10 | LP[IPV][ASG]A[TM]AMAST[TA]S[AST]AA[ST][MF][LV][LM][SV][GD]S[SM][STNP][SP][AHQS][DGLS][GNQ] | Low complexity region |
