# Supplementary material for: Identification of Camellia oleifera WRKY transcription factor genes and functional characterization of CoWRKY78
Source: Front Plant Sci. 2023 Mar 9;14:1110366. doi: 10.3389/fpls.2023.1110366 (PMC10036053; doi:10.3389/fpls.2023.1110366)
Supplement: Supplementary file 11 [file Table_4.docx]

**TABLE S4. Numbers of *WRKY* genes from different origins in *A. thaliana*, *P. trichocarpa* and *C. oleifera* genomes.**

|  | Number of WRKY genes | Number of genes from different origins (percentage) | | | |
| --- | --- | --- | --- | --- | --- |
|  |  | WGD/Segmental | Tandem | Proximal | Dispersed |
| *A. thaliana* | 72 | 30 (41.7%) | 2 (2.8%) | 2 (2.8%) | 38 (52.7%) |
| *P. trichocarpa* | 97 | 90 (92.8%) | 1 (1.0%) | 0 (0.0%) | 6 (6.2%) |
| *C. oleifera* | 91 | 57 (62.6%) | 2 (2.2%) | 6 (6.6%) | 26 (28.6%) |
