# Supplementary material for: Identification of Camellia oleifera WRKY transcription factor genes and functional characterization of CoWRKY78
Source: Front Plant Sci. 2023 Mar 9;14:1110366. doi: 10.3389/fpls.2023.1110366 (PMC10036053; doi:10.3389/fpls.2023.1110366)
Supplement: Supplementary file 12 [file Table_5.docx]

**TABLE S5. Orthologous gene pairs of *WRKYs* between *C. oleifera* and *A. thaliana*.**

| Collinear gene pairs | | | | Ka | Ks | Ka/Ks |
| --- | --- | --- | --- | --- | --- | --- |
| *C. oleifera* | Chr name | *A. thaliana* | Chr name |  |  |  |
| CoWRKY3 | Chr1 | AT2G44745.1 | Chr2 | 0.356163 | 3.880294 | 0.091788 |
| CoWRKY5 | Chr1 | AT2G46130.1 | Chr2 | 0.490123 | NaN | NaN |
| CoWRKY6 | Chr1 | AT2G46400.1 | Chr2 | 0.493212 | 2.81817 | 0.175012 |
| CoWRKY7 | Chr2 | AT2G47260.1 | Chr2 | 0.418574 | 2.206432 | 0.189706 |
| CoWRKY7 | Chr2 | AT5G49520.1 | Chr5 | 0.351926 | NaN | NaN |
| CoWRKY11 | Chr2 | AT4G26440.1 | Chr4 | 0.363101 | 2.510886 | 0.144611 |
| CoWRKY11 | Chr2 | AT5G56270.1 | Chr5 | 0.357889 | 3.078109 | 0.116269 |
| CoWRKY15 | Chr3 | AT2G30250.1 | Chr2 | 0.343769 | 1.970553 | 0.174453 |
| CoWRKY16 | Chr3 | AT2G37260.1 | Chr2 | 0.364414 | 1.893767 | 0.192428 |
| CoWRKY18 | Chr3 | AT5G01900.1 | Chr5 | 0.747199 | NaN | NaN |
| CoWRKY20 | Chr4 | AT1G29860.1 | Chr1 | 0.363827 | 2.111668 | 0.172294 |
| CoWRKY20 | Chr4 | AT4G18170.1 | Chr4 | 0.38372 | NaN | NaN |
| CoWRKY20 | Chr4 | AT5G46350.1 | Chr5 | 0.455542 | NaN | NaN |
| CoWRKY21 | Chr4 | AT1G29280.1 | Chr1 | 0.446675 | NaN | NaN |
| CoWRKY22 | Chr4 | AT1G62300.1 | Chr1 | 0.320593 | 2.745135 | 0.116786 |
| CoWRKY22 | Chr4 | AT4G04450.1 | Chr4 | 0.354719 | NaN | NaN |
| CoWRKY22 | Chr4 | AT4G22070.1 | Chr4 | 0.338559 | 3.60076 | 0.094024 |
| CoWRKY25 | Chr6 | AT2G47260.1 | Chr2 | 0.37979 | 1.667436 | 0.227769 |
| CoWRKY25 | Chr6 | AT3G62340.1 | Chr3 | 0.398439 | 1.543063 | 0.258213 |
| CoWRKY28 | Chr7 | AT3G01970.1 | Chr3 | 0.26592 | 1.667825 | 0.159441 |
| CoWRKY29 | Chr7 | AT1G80840.1 | Chr1 | 0.318679 | 2.837808 | 0.112298 |
| CoWRKY30 | Chr7 | AT1G18860.1 | Chr1 | 0.435085 | 1.670004 | 0.26053 |
| CoWRKY30 | Chr7 | AT1G69810.1 | Chr1 | 0.524984 | 2.599637 | 0.201945 |
| CoWRKY30 | Chr7 | AT5G15130.1 | Chr5 | 0.484918 | 1.630074 | 0.297482 |
| CoWRKY31 | Chr7 | AT2G23320.1 | Chr2 | 0.371279 | NaN | NaN |
| CoWRKY34 | Chr8 | AT2G47260.1 | Chr2 | 0.39581 | 3.026046 | 0.130801 |
| CoWRKY34 | Chr8 | AT3G62340.1 | Chr3 | 0.480171 | 1.591702 | 0.301671 |
| CoWRKY36 | Chr8 | AT2G30590.1 | Chr2 | 0.259361 | 2.227532 | 0.116434 |
| CoWRKY38 | Chr8 | AT2G30250.1 | Chr2 | 0.344945 | 2.229436 | 0.154723 |
| CoWRKY39 | Chr9 | AT1G80840.1 | Chr1 | 0.291017 | 2.506539 | 0.116103 |
| CoWRKY44 | Chr10 | AT2G40740.3 | Chr2 | 0.465416 | 2.690294 | 0.172998 |
| CoWRKY44 | Chr10 | AT3G56400.1 | Chr3 | 0.789256 | 2.798366 | 0.282042 |
| CoWRKY45 | Chr10 | AT2G40750.1 | Chr2 | 0.539663 | 1.547812 | 0.348662 |
| CoWRKY45 | Chr10 | AT3G56400.1 | Chr3 | 0.540306 | 3.644859 | 0.148238 |
| CoWRKY47 | Chr10 | AT5G52830.1 | Chr5 | 0.545805 | 2.569923 | 0.212382 |
| CoWRKY50 | Chr10 | AT4G39410.1 | Chr4 | 0.518823 | NaN | NaN |
| CoWRKY51 | Chr10 | AT2G23320.1 | Chr2 | 0.33552 | 3.041656 | 0.110308 |
| CoWRKY52 | Chr10 | AT1G69810.1 | Chr1 | 0.595109 | 1.541904 | 0.385957 |
| CoWRKY52 | Chr10 | AT5G15130.1 | Chr5 | 0.411084 | 2.172311 | 0.189238 |
| CoWRKY53 | Chr10 | AT5G15130.1 | Chr5 | 0.412656 | 1.980801 | 0.208328 |
| CoWRKY54 | Chr10 | AT3G01970.1 | Chr3 | 0.240359 | NaN | NaN |
| CoWRKY56 | Chr11 | AT2G40750.1 | Chr2 | 0.566748 | 1.722514 | 0.329023 |
| CoWRKY57 | Chr11 | AT2G40740.3 | Chr2 | 0.396603 | 1.73399 | 0.228723 |
| CoWRKY61 | Chr11 | AT5G56270.1 | Chr5 | 0.314531 | 2.723021 | 0.115508 |
| CoWRKY62 | Chr11 | AT4G26440.1 | Chr4 | 0.372493 | 2.111064 | 0.176448 |
| CoWRKY62 | Chr11 | AT5G56270.1 | Chr5 | 0.318551 | 2.894753 | 0.110044 |
| CoWRKY64 | Chr12 | AT5G13080.1 | Chr5 | 0.25253 | 4.24793 | 0.059448 |
| CoWRKY65 | Chr12 | AT1G69310.2 | Chr1 | 0.405308 | NaN | NaN |
| CoWRKY66 | Chr12 | AT1G13960.1 | Chr1 | 0.300607 | 2.951366 | 0.101854 |
| CoWRKY66 | Chr12 | AT2G03340.1 | Chr2 | 0.29138 | 3.687793 | 0.079012 |
| CoWRKY67 | Chr12 | AT3G04670.1 | Chr3 | 0.298007 | 1.978304 | 0.150638 |
| CoWRKY68 | Chr12 | AT2G46400.1 | Chr2 | 0.550061 | 3.425682 | 0.16057 |
| CoWRKY68 | Chr12 | AT4G23810.1 | Chr4 | 0.458531 | NaN | NaN |
| CoWRKY69 | Chr12 | AT4G01250.1 | Chr4 | 0.377102 | 1.730041 | 0.217973 |
| CoWRKY69 | Chr12 | AT4G23550.1 | Chr4 | 0.563423 | 2.090604 | 0.269503 |
| CoWRKY71 | Chr12 | AT4G30935.1 | Chr4 | 0.371276 | 3.28932 | 0.112873 |
| CoWRKY73 | Chr13 | AT5G13080.1 | Chr5 | 0.29531 | NaN | NaN |
| CoWRKY75 | Chr13 | AT2G37260.1 | Chr2 | 0.417609 | 3.449828 | 0.121052 |
| CoWRKY78 | Chr13 | AT5G01900.1 | Chr5 | 0.692284 | NaN | NaN |
| CoWRKY80 | Chr14 | AT4G11070.1 | Chr4 | 0.403029 | 2.795014 | 0.144196 |
| CoWRKY80 | Chr14 | AT4G23810.1 | Chr4 | 0.396919 | 3.372581 | 0.11769 |
| CoWRKY81 | Chr14 | AT4G23550.1 | Chr4 | 0.496456 | NaN | NaN |
| CoWRKY83 | Chr14 | AT2G25000.1 | Chr2 | 0.547766 | 2.69627 | 0.203157 |
| CoWRKY83 | Chr14 | AT4G31800.1 | Chr4 | 0.509323 | 3.814434 | 0.133525 |
| CoWRKY84 | Chr14 | AT2G24570.1 | Chr2 | 0.242727 | 1.69683 | 0.143048 |
| CoWRKY84 | Chr14 | AT4G31550.1 | Chr4 | 0.258461 | 2.53506 | 0.101954 |
| CoWRKY86 | Chr15 | AT2G46400.1 | Chr2 | 0.624852 | 3.80705 | 0.16413 |
| CoWRKY86 | Chr15 | AT4G11070.1 | Chr4 | 0.386527 | 3.425505 | 0.112838 |
| CoWRKY86 | Chr15 | AT4G23810.1 | Chr4 | 0.416139 | NaN | NaN |
| CoWRKY88 | Chr15 | AT1G64000.1 | Chr1 | 0.588893 | 1.444762 | 0.407606 |
| CoWRKY88 | Chr15 | AT2G46130.1 | Chr2 | 0.35935 | 3.391968 | 0.105942 |
| CoWRKY88 | Chr15 | AT5G41570.1 | Chr5 | 0.679331 | 1.437043 | 0.472728 |
| CoWRKY89 | Chr15 | AT4G23550.1 | Chr4 | 0.5503 | 2.412511 | 0.228103 |
| CoWRKY90 | Chr15 | AT1G69310.2 | Chr1 | 0.392104 | 3.756379 | 0.104384 |
