# Supplementary material for: Identification of Camellia oleifera WRKY transcription factor genes and functional characterization of CoWRKY78
Source: Front Plant Sci. 2023 Mar 9;14:1110366. doi: 10.3389/fpls.2023.1110366 (PMC10036053; doi:10.3389/fpls.2023.1110366)
Supplement: Supplementary file 13 [file Table_6.docx]

**TABLE S6. Orthologous gene pairs of *WRKYs* between *C. oleifera* and *P. trichocarpa*.**

| Collinear gene pairs | | | | Ka | Ks | Ka/Ks |
| --- | --- | --- | --- | --- | --- | --- |
| *C. oleifera* | Chr name | *P. trichocarpa* | Chr name |  |  |  |
| CoWRKY1 | Chr1 | Potri.001G208600.1 | Chr1 | 0.293465 | 1.317142 | 0.222805 |
| CoWRKY3 | Chr1 | Potri.002G138900.1 | Chr2 | 0.18096 | 1.077488 | 0.167946 |
| CoWRKY3 | Chr1 | Potri.014G050000.1 | Chr14 | 0.195679 | 0.866293 | 0.225881 |
| CoWRKY4 | Chr1 | Potri.001G099001.1 | Chr1 | 0.501787 | 3.45592 | 0.145196 |
| CoWRKY4 | Chr1 | Potri.002G164400.4 | Chr2 | 0.314226 | 1.138674 | 0.275958 |
| CoWRKY4 | Chr1 | Potri.003G132700.1 | Chr3 | 0.534554 | NaN | NaN |
| CoWRKY4 | Chr1 | Potri.014G090300.1 | Chr14 | 0.319013 | 1.296394 | 0.246077 |
| CoWRKY5 | Chr1 | Potri.002G164900.1 | Chr2 | 0.369934 | 2.077928 | 0.17803 |
| CoWRKY5 | Chr1 | Potri.014G090700.1 | Chr14 | 0.381378 | 2.517533 | 0.151489 |
| CoWRKY6 | Chr1 | Potri.001G092900.1 | Chr1 | 0.425388 | 1.981961 | 0.21463 |
| CoWRKY6 | Chr1 | Potri.002G168700.1 | Chr2 | 0.320565 | 1.744827 | 0.183723 |
| CoWRKY6 | Chr1 | Potri.003G138600.1 | Chr3 | 0.413936 | 2.591448 | 0.159732 |
| CoWRKY6 | Chr1 | Potri.014G096200.1 | Chr14 | 0.285626 | 1.549374 | 0.184349 |
| CoWRKY7 | Chr2 | Potri.002G193000.1 | Chr2 | 0.324218 | 2.362308 | 0.137246 |
| CoWRKY7 | Chr2 | Potri.008G103300.1 | Chr8 | 0.235929 | 1.390465 | 0.169676 |
| CoWRKY7 | Chr2 | Potri.010G147700.1 | Chr10 | 0.214875 | 1.275472 | 0.168467 |
| CoWRKY7 | Chr2 | Potri.014G118200.1 | Chr14 | 0.360935 | 2.218823 | 0.16267 |
| CoWRKY8 | Chr2 | Potri.014G155100.1 | Chr14 | 0.217021 | 1.746845 | 0.124236 |
| CoWRKY9 | Chr2 | Potri.002G221600.1 | Chr2 | 0.36405 | 1.265746 | 0.287617 |
| CoWRKY9 | Chr2 | Potri.014G164300.6 | Chr14 | 0.370856 | 1.011692 | 0.36657 |
| CoWRKY10 | Chr2 | Potri.001G361600.2 | Chr1 | 0.240532 | 0.977493 | 0.24607 |
| CoWRKY11 | Chr2 | Potri.001G472800.9 | Chr1 | 0.202039 | 1.016622 | 0.198735 |
| CoWRKY11 | Chr2 | Potri.011G169300.1 | Chr11 | 0.212354 | 0.994348 | 0.213561 |
| CoWRKY12 | Chr3 | Potri.002G123300.1 | Chr2 | 0.241265 | 2.779302 | 0.086808 |
| CoWRKY12 | Chr3 | Potri.005G141400.2 | Chr5 | 0.239255 | NaN | NaN |
| CoWRKY12 | Chr3 | Potri.007G047400.1 | Chr7 | 0.22963 | NaN | NaN |
| CoWRKY12 | Chr3 | Potri.014G024200.2 | Chr14 | 0.208142 | 2.649959 | 0.078545 |
| CoWRKY14 | Chr3 | Potri.019G123500.3 | Chr19 | 0.213023 | 1.430735 | 0.148891 |
| CoWRKY15 | Chr3 | Potri.006G105300.1 | Chr6 | 0.303331 | 1.557554 | 0.194748 |
| CoWRKY15 | Chr3 | Potri.013G153400.1 | Chr13 | 0.213653 | 1.272829 | 0.167856 |
| CoWRKY15 | Chr3 | Potri.016G128300.1 | Chr16 | 0.291798 | 1.830612 | 0.159399 |
| CoWRKY16 | Chr3 | Potri.006G133200.11 | Chr6 | 0.205304 | 0.801943 | 0.256009 |
| CoWRKY16 | Chr3 | Potri.016G083600.6 | Chr16 | 0.223403 | 0.939152 | 0.237878 |
| CoWRKY18 | Chr3 | Potri.006G109100.1 | Chr6 | 0.359138 | 2.433604 | 0.147574 |
| CoWRKY18 | Chr3 | Potri.016G137900.1 | Chr16 | 0.347527 | 2.818542 | 0.1233 |
| CoWRKY19 | Chr4 | Potri.011G157100.1 | Chr11 | 0.238392 | 1.287982 | 0.185089 |
| CoWRKY20 | Chr4 | Potri.001G352400.1 | Chr1 | 0.267232 | 1.413851 | 0.18901 |
| CoWRKY20 | Chr4 | Potri.002G059100.1 | Chr2 | 0.317676 | 2.518303 | 0.126147 |
| CoWRKY20 | Chr4 | Potri.011G079300.2 | Chr11 | 0.265521 | 1.448672 | 0.183286 |
| CoWRKY21 | Chr4 | Potri.004G060900.1 | Chr4 | 0.321828 | 1.714714 | 0.187686 |
| CoWRKY21 | Chr4 | Potri.011G070100.1 | Chr11 | 0.329525 | 3.71157 | 0.088783 |
| CoWRKY22 | Chr4 | Potri.004G007500.1 | Chr4 | 0.213334 | 1.54831 | 0.137785 |
| CoWRKY22 | Chr4 | Potri.011G007800.1 | Chr11 | 0.222127 | 1.713367 | 0.129644 |
| CoWRKY22 | Chr4 | Potri.014G155100.1 | Chr14 | 0.303465 | 1.831398 | 0.165701 |
| CoWRKY24 | Chr5 | Potri.002G193000.1 | Chr2 | 0.322778 | 1.947686 | 0.165724 |
| CoWRKY24 | Chr5 | Potri.008G103300.1 | Chr8 | 0.23898 | 1.406956 | 0.169856 |
| CoWRKY24 | Chr5 | Potri.010G147700.1 | Chr10 | 0.219845 | 1.299621 | 0.169161 |
| CoWRKY24 | Chr5 | Potri.014G118200.1 | Chr14 | 0.328718 | 2.502454 | 0.131358 |
| CoWRKY25 | Chr6 | Potri.002G193000.1 | Chr2 | 0.32341 | 1.071295 | 0.301887 |
| CoWRKY25 | Chr6 | Potri.014G118200.1 | Chr14 | 0.331677 | 1.001073 | 0.331321 |
| CoWRKY26 | Chr6 | Potri.002G195300.2 | Chr2 | 0.269643 | 1.153583 | 0.233744 |
| CoWRKY26 | Chr6 | Potri.014G119800.1 | Chr14 | 0.255669 | 0.916229 | 0.279045 |
| CoWRKY27 | Chr7 | Potri.017G066860.1 | Chr17 | 0.298214 | 1.229172 | 0.242614 |
| CoWRKY28 | Chr7 | Potri.001G328000.2 | Chr1 | 0.293626 | 1.420319 | 0.206733 |
| CoWRKY28 | Chr7 | Potri.015G099200.1 | Chr15 | 0.385604 | 1.645539 | 0.234333 |
| CoWRKY28 | Chr7 | Potri.017G066860.1 | Chr17 | 0.29867 | 1.365428 | 0.218737 |
| CoWRKY29 | Chr7 | Potri.001G044500.1 | Chr1 | 0.216844 | 1.103266 | 0.196547 |
| CoWRKY29 | Chr7 | Potri.003G182200.2 | Chr3 | 0.202677 | 1.48524 | 0.136461 |
| CoWRKY30 | Chr7 | Potri.015G064100.1 | Chr15 | 0.344002 | 1.173173 | 0.293223 |
| CoWRKY30 | Chr7 | Potri.017G079500.1 | Chr17 | 0.395147 | 1.681802 | 0.234955 |
| CoWRKY32 | Chr7 | Potri.002G123300.1 | Chr2 | 0.285515 | 2.392015 | 0.119362 |
| CoWRKY32 | Chr7 | Potri.005G141400.2 | Chr5 | 0.205825 | NaN | NaN |
| CoWRKY32 | Chr7 | Potri.007G047400.1 | Chr7 | 0.178959 | NaN | NaN |
| CoWRKY32 | Chr7 | Potri.014G024200.2 | Chr14 | 0.309939 | 2.23087 | 0.138932 |
| CoWRKY33 | Chr7 | Potri.006G184800.2 | Chr6 | 0.234595 | 1.048028 | 0.223844 |
| CoWRKY33 | Chr7 | Potri.018G107000.5 | Chr18 | 0.245504 | 0.994938 | 0.246753 |
| CoWRKY34 | Chr8 | Potri.002G193000.1 | Chr2 | 0.296051 | 1.062448 | 0.27865 |
| CoWRKY34 | Chr8 | Potri.008G103300.1 | Chr8 | 0.370606 | 2.868126 | 0.129215 |
| CoWRKY34 | Chr8 | Potri.010G147700.1 | Chr10 | 0.383565 | 2.467394 | 0.155453 |
| CoWRKY34 | Chr8 | Potri.014G118200.1 | Chr14 | 0.296693 | 1.243267 | 0.238639 |
| CoWRKY35 | Chr8 | Potri.002G195300.2 | Chr2 | 0.281048 | 0.835335 | 0.336449 |
| CoWRKY35 | Chr8 | Potri.014G119800.1 | Chr14 | 0.246613 | 0.842253 | 0.292802 |
| CoWRKY36 | Chr8 | Potri.002G043500.1 | Chr2 | 0.135003 | 1.256705 | 0.107426 |
| CoWRKY36 | Chr8 | Potri.005G219500.1 | Chr5 | 0.135825 | 1.246636 | 0.108954 |
| CoWRKY36 | Chr8 | Potri.013G042600.1 | Chr13 | 0.240613 | 1.381795 | 0.174131 |
| CoWRKY38 | Chr8 | Potri.006G105300.1 | Chr6 | 0.275029 | 2.23735 | 0.122926 |
| CoWRKY38 | Chr8 | Potri.013G153400.1 | Chr13 | 0.189877 | 1.694537 | 0.112052 |
| CoWRKY38 | Chr8 | Potri.016G128300.1 | Chr16 | 0.252815 | 2.436956 | 0.103742 |
| CoWRKY38 | Chr8 | Potri.019G123500.3 | Chr19 | 0.182447 | 1.641882 | 0.11112 |
| CoWRKY39 | Chr9 | Potri.001G044500.1 | Chr1 | 0.230513 | 1.14563 | 0.201211 |
| CoWRKY39 | Chr9 | Potri.003G182200.2 | Chr3 | 0.222715 | 1.011589 | 0.220163 |
| CoWRKY39 | Chr9 | Potri.006G263600.1 | Chr6 | 0.330391 | 1.620524 | 0.203879 |
| CoWRKY39 | Chr9 | Potri.018G019700.1 | Chr18 | 0.597874 | 2.456704 | 0.243364 |
| CoWRKY40 | Chr9 | Potri.001G044500.1 | Chr1 | 0.230638 | 1.141292 | 0.202085 |
| CoWRKY40 | Chr9 | Potri.003G182200.2 | Chr3 | 0.224687 | 1.008183 | 0.222864 |
| CoWRKY41 | Chr9 | Potri.015G064100.1 | Chr15 | 0.336199 | 1.021394 | 0.329157 |
| CoWRKY41 | Chr9 | Potri.017G079500.1 | Chr17 | 0.362239 | 1.149009 | 0.315262 |
| CoWRKY42 | Chr10 | Potri.013G086000.1 | Chr13 | 0.373836 | 1.063178 | 0.351622 |
| CoWRKY44 | Chr10 | Potri.013G090400.1 | Chr13 | 0.322794 | 1.951596 | 0.1654 |
| CoWRKY44 | Chr10 | Potri.019G059300.2 | Chr19 | 0.341411 | 1.734034 | 0.196888 |
| CoWRKY45 | Chr10 | Potri.006G109100.1 | Chr6 | 0.456106 | 1.578278 | 0.28899 |
| CoWRKY45 | Chr10 | Potri.013G090300.1 | Chr13 | 0.368199 | 1.173537 | 0.313751 |
| CoWRKY45 | Chr10 | Potri.016G137900.1 | Chr16 | 0.48492 | 1.585823 | 0.305784 |
| CoWRKY46 | Chr10 | Potri.003G132700.1 | Chr3 | 0.611412 | NaN | NaN |
| CoWRKY46 | Chr10 | Potri.004G072000.1 | Chr4 | 0.455947 | 2.543947 | 0.179228 |
| CoWRKY46 | Chr10 | Potri.017G149000.1 | Chr17 | 0.464522 | 1.730974 | 0.268359 |
| CoWRKY47 | Chr10 | Potri.001G099001.1 | Chr1 | 0.614003 | 2.795733 | 0.219621 |
| CoWRKY49 | Chr10 | Potri.001G092900.1 | Chr1 | 0.393509 | 1.752314 | 0.224565 |
| CoWRKY49 | Chr10 | Potri.002G168700.1 | Chr2 | 0.375827 | 2.36655 | 0.158808 |
| CoWRKY49 | Chr10 | Potri.003G138600.1 | Chr3 | 0.376288 | 1.686746 | 0.223085 |
| CoWRKY49 | Chr10 | Potri.012G031700.1 | Chr12 | 0.352043 | 1.160144 | 0.303448 |
| CoWRKY49 | Chr10 | Potri.014G096200.1 | Chr14 | 0.374191 | 2.382985 | 0.157026 |
| CoWRKY50 | Chr10 | Potri.005G086400.1 | Chr5 | 0.18217 | 0.717755 | 0.253805 |
| CoWRKY50 | Chr10 | Potri.007G078200.2 | Chr7 | 0.204608 | 0.689353 | 0.296812 |
| CoWRKY51 | Chr10 | Potri.002G123300.1 | Chr2 | 0.305021 | 1.888308 | 0.161531 |
| CoWRKY51 | Chr10 | Potri.005G141400.2 | Chr5 | 0.206098 | 2.105361 | 0.097892 |
| CoWRKY51 | Chr10 | Potri.007G047400.1 | Chr7 | 0.192046 | 1.871742 | 0.102603 |
| CoWRKY51 | Chr10 | Potri.014G024200.2 | Chr14 | 0.269098 | 2.514331 | 0.107026 |
| CoWRKY53 | Chr10 | Potri.017G079500.1 | Chr17 | 0.321988 | 1.145335 | 0.28113 |
| CoWRKY54 | Chr10 | Potri.001G058800.1 | Chr1 | 0.229843 | 2.309873 | 0.099505 |
| CoWRKY54 | Chr10 | Potri.001G328000.2 | Chr1 | 0.249858 | 1.276058 | 0.195805 |
| CoWRKY54 | Chr10 | Potri.003G169100.1 | Chr3 | 0.243055 | 1.862437 | 0.130504 |
| CoWRKY54 | Chr10 | Potri.015G099200.1 | Chr15 | 0.393075 | 1.396379 | 0.281496 |
| CoWRKY54 | Chr10 | Potri.017G066860.1 | Chr17 | 0.263206 | 1.397552 | 0.188334 |
| CoWRKY55 | Chr11 | Potri.001G092900.1 | Chr1 | 0.325468 | 3.729589 | 0.087266 |
| CoWRKY55 | Chr11 | Potri.002G168700.1 | Chr2 | 0.345717 | 2.729624 | 0.126654 |
| CoWRKY55 | Chr11 | Potri.012G031700.1 | Chr12 | 0.302556 | 1.348967 | 0.224287 |
| CoWRKY56 | Chr11 | Potri.013G090300.1 | Chr13 | 0.357952 | 0.887426 | 0.40336 |
| CoWRKY56 | Chr11 | Potri.016G137900.1 | Chr16 | 0.473729 | 1.579299 | 0.299962 |
| CoWRKY57 | Chr11 | Potri.013G090400.1 | Chr13 | 0.352088 | 1.776379 | 0.198205 |
| CoWRKY57 | Chr11 | Potri.019G059300.2 | Chr19 | 0.333895 | 1.17239 | 0.284798 |
| CoWRKY58 | Chr11 | Potri.002G228400.1 | Chr2 | 0.277157 | 2.050215 | 0.135185 |
| CoWRKY58 | Chr11 | Potri.014G155100.1 | Chr14 | 0.244043 | 1.89582 | 0.128727 |
| CoWRKY59 | Chr11 | Potri.001G361600.2 | Chr1 | 0.518606 | 1.732786 | 0.299291 |
| CoWRKY59 | Chr11 | Potri.002G221600.1 | Chr2 | 0.368748 | 1.30584 | 0.282384 |
| CoWRKY59 | Chr11 | Potri.014G164300.6 | Chr14 | 0.409367 | 1.093303 | 0.374431 |
| CoWRKY60 | Chr11 | Potri.004G007500.1 | Chr4 | 0.328731 | 1.577832 | 0.208343 |
| CoWRKY60 | Chr11 | Potri.014G155100.1 | Chr14 | 0.326338 | 1.690253 | 0.19307 |
| CoWRKY61 | Chr11 | Potri.001G472800.9 | Chr1 | 0.183623 | 0.92686 | 0.198113 |
| CoWRKY61 | Chr11 | Potri.011G169300.1 | Chr11 | 0.190851 | 0.979003 | 0.194944 |
| CoWRKY62 | Chr11 | Potri.001G472800.9 | Chr1 | 0.178447 | 0.961601 | 0.185573 |
| CoWRKY62 | Chr11 | Potri.011G169300.1 | Chr11 | 0.184089 | 1.003065 | 0.183526 |
| CoWRKY64 | Chr12 | Potri.001G058800.1 | Chr1 | 0.162852 | 0.999569 | 0.162922 |
| CoWRKY64 | Chr12 | Potri.001G328000.2 | Chr1 | 0.205812 | 1.157024 | 0.17788 |
| CoWRKY64 | Chr12 | Potri.003G169100.1 | Chr3 | 0.158364 | 1.313816 | 0.120537 |
| CoWRKY64 | Chr12 | Potri.015G099200.1 | Chr15 | 0.313554 | 1.100614 | 0.28489 |
| CoWRKY65 | Chr12 | Potri.008G094000.1 | Chr8 | 0.293738 | 1.302878 | 0.225453 |
| CoWRKY65 | Chr12 | Potri.010G160100.5 | Chr10 | 0.283347 | 1.338606 | 0.211673 |
| CoWRKY66 | Chr12 | Potri.008G091900.1 | Chr8 | 0.27128 | 1.344383 | 0.201788 |
| CoWRKY66 | Chr12 | Potri.010G163000.1 | Chr10 | 0.253791 | 1.452239 | 0.174759 |
| CoWRKY66 | Chr12 | Potri.017G088300.3 | Chr17 | 0.381059 | 1.388833 | 0.274374 |
| CoWRKY67 | Chr12 | Potri.005G055300.1 | Chr5 | 0.178329 | 0.862952 | 0.20665 |
| CoWRKY67 | Chr12 | Potri.013G042600.1 | Chr13 | 0.182505 | 0.916393 | 0.199156 |
| CoWRKY68 | Chr12 | Potri.001G092900.1 | Chr1 | 0.378957 | 1.650854 | 0.229552 |
| CoWRKY68 | Chr12 | Potri.002G168700.1 | Chr2 | 0.270919 | 1.785153 | 0.151763 |
| CoWRKY68 | Chr12 | Potri.003G138600.1 | Chr3 | 0.369228 | 2.252747 | 0.163901 |
| CoWRKY68 | Chr12 | Potri.014G096200.1 | Chr14 | 0.266397 | 1.925993 | 0.138317 |
| CoWRKY69 | Chr12 | Potri.001G099001.1 | Chr1 | 0.514938 | 3.016195 | 0.170724 |
| CoWRKY69 | Chr12 | Potri.002G164400.4 | Chr2 | 0.261123 | 1.409078 | 0.185315 |
| CoWRKY69 | Chr12 | Potri.003G132700.1 | Chr3 | 0.489202 | NaN | NaN |
| CoWRKY69 | Chr12 | Potri.014G090300.1 | Chr14 | 0.253365 | 1.363361 | 0.185838 |
| CoWRKY71 | Chr12 | Potri.006G184800.2 | Chr6 | 0.297189 | 1.097449 | 0.2708 |
| CoWRKY71 | Chr12 | Potri.018G107000.5 | Chr18 | 0.268263 | 0.998524 | 0.26866 |
| CoWRKY73 | Chr13 | Potri.001G058800.1 | Chr1 | 0.131868 | 1.11416 | 0.118357 |
| CoWRKY73 | Chr13 | Potri.001G328000.2 | Chr1 | 0.214477 | 1.31767 | 0.16277 |
| CoWRKY73 | Chr13 | Potri.003G169100.1 | Chr3 | 0.144209 | 1.254576 | 0.114946 |
| CoWRKY73 | Chr13 | Potri.015G099200.1 | Chr15 | 0.299117 | 0.922286 | 0.324322 |
| CoWRKY74 | Chr13 | Potri.006G263600.1 | Chr6 | 0.495407 | NaN | NaN |
| CoWRKY74 | Chr13 | Potri.018G019700.1 | Chr18 | 0.445558 | 1.863048 | 0.239155 |
| CoWRKY75 | Chr13 | Potri.006G133200.11 | Chr6 | 0.231532 | 0.83201 | 0.27828 |
| CoWRKY75 | Chr13 | Potri.016G083600.6 | Chr16 | 0.251032 | 0.870019 | 0.288536 |
| CoWRKY76 | Chr13 | Potri.006G087000.1 | Chr6 | 0.296406 | 1.463249 | 0.202567 |
| CoWRKY76 | Chr13 | Potri.016G099900.1 | Chr16 | 0.31325 | 1.470955 | 0.212957 |
| CoWRKY77 | Chr13 | Potri.006G105300.1 | Chr6 | 0.265463 | 1.078147 | 0.246222 |
| CoWRKY77 | Chr13 | Potri.013G153400.1 | Chr13 | 0.279646 | 1.94626 | 0.143684 |
| CoWRKY77 | Chr13 | Potri.016G128300.1 | Chr16 | 0.256228 | 1.054756 | 0.242926 |
| CoWRKY77 | Chr13 | Potri.019G123500.3 | Chr19 | 0.288234 | 1.86234 | 0.15477 |
| CoWRKY78 | Chr13 | Potri.006G109100.1 | Chr6 | 0.284035 | NaN | NaN |
| CoWRKY78 | Chr13 | Potri.016G137900.1 | Chr16 | 0.313569 | 2.380655 | 0.131715 |
| CoWRKY80 | Chr14 | Potri.001G092900.1 | Chr1 | 0.312439 | 1.220558 | 0.25598 |
| CoWRKY80 | Chr14 | Potri.002G168700.1 | Chr2 | 0.381624 | NaN | NaN |
| CoWRKY80 | Chr14 | Potri.003G138600.1 | Chr3 | 0.313191 | 1.172585 | 0.267095 |
| CoWRKY80 | Chr14 | Potri.014G096200.1 | Chr14 | 0.355515 | 2.446013 | 0.145345 |
| CoWRKY81 | Chr14 | Potri.001G099001.1 | Chr1 | 0.4017 | 1.121762 | 0.358097 |
| CoWRKY81 | Chr14 | Potri.002G164400.4 | Chr2 | 0.432182 | 2.648035 | 0.163208 |
| CoWRKY81 | Chr14 | Potri.003G132700.1 | Chr3 | 0.401412 | 1.008783 | 0.397917 |
| CoWRKY81 | Chr14 | Potri.014G090300.1 | Chr14 | 0.417761 | 3.109898 | 0.134333 |
| CoWRKY83 | Chr14 | Potri.003G182200.2 | Chr3 | 0.373161 | 1.181812 | 0.315753 |
| CoWRKY83 | Chr14 | Potri.006G263600.1 | Chr6 | 0.36656 | 1.441004 | 0.254378 |
| CoWRKY83 | Chr14 | Potri.018G019700.1 | Chr18 | 0.482896 | 2.576912 | 0.187393 |
| CoWRKY84 | Chr14 | Potri.018G008500.1 | Chr18 | 0.226304 | 1.651452 | 0.137033 |
| CoWRKY85 | Chr14 | Potri.006G224100.1 | Chr6 | 0.22714 | 3.280874 | 0.069232 |
| CoWRKY86 | Chr15 | Potri.001G092900.1 | Chr1 | 0.261681 | 1.700695 | 0.153867 |
| CoWRKY86 | Chr15 | Potri.002G168700.1 | Chr2 | 0.350668 | NaN | NaN |
| CoWRKY86 | Chr15 | Potri.003G138600.1 | Chr3 | 0.256443 | 1.630361 | 0.157292 |
| CoWRKY86 | Chr15 | Potri.012G031700.1 | Chr12 | 0.396225 | 2.970741 | 0.133376 |
| CoWRKY86 | Chr15 | Potri.014G096200.1 | Chr14 | 0.333955 | 2.105332 | 0.158623 |
| CoWRKY87 | Chr15 | Potri.001G099001.1 | Chr1 | 0.437112 | 1.36267 | 0.320776 |
| CoWRKY87 | Chr15 | Potri.002G164400.4 | Chr2 | 0.46434 | 1.604708 | 0.289361 |
| CoWRKY87 | Chr15 | Potri.003G132700.1 | Chr3 | 0.455133 | 1.305905 | 0.348519 |
| CoWRKY87 | Chr15 | Potri.014G090300.1 | Chr14 | 0.465256 | 1.760134 | 0.26433 |
| CoWRKY88 | Chr15 | Potri.014G090700.1 | Chr14 | 0.394099 | 1.481829 | 0.265954 |
| CoWRKY89 | Chr15 | Potri.001G099001.1 | Chr1 | 0.442573 | 1.47701 | 0.299641 |
| CoWRKY89 | Chr15 | Potri.014G090300.1 | Chr14 | 0.467882 | 1.762939 | 0.265399 |
| CoWRKY90 | Chr15 | Potri.008G094000.1 | Chr8 | 0.241033 | 1.036651 | 0.232511 |
| CoWRKY90 | Chr15 | Potri.010G160100.5 | Chr10 | 0.24955 | 1.238279 | 0.20153 |
