# Supplementary material for: Identification of Camellia oleifera WRKY transcription factor genes and functional characterization of CoWRKY78
Source: Front Plant Sci. 2023 Mar 9;14:1110366. doi: 10.3389/fpls.2023.1110366 (PMC10036053; doi:10.3389/fpls.2023.1110366)
Supplement: Supplementary file 14 [file Table_7.docx]

**TABLE S7. The number of W-box in the 5’ regulatory sequences of tobacco stress-related genes.**

| W-box | Stress-related genes | | | | | |
| --- | --- | --- | --- | --- | --- | --- |
|  | *NtPOD* | *NtSOD* | *NtPAL* | *NtPR1* | *NtNPR1* | *NtPDF1.2* |
| TGAC(C/T) | 7 | 6 | 5 | 10 | 3 | 2 |
| TTGAC | 4 | 3 | 3 | 1 | 5 | 4 |
